# Predicting Cancer Drug Response In Vivo by Learning an Optimal Feature Selection of Tumour Molecular Profiles

**DOI:** 10.3390/biomedicines9101319

**Published:** 2021-09-26

**Authors:** Linh C. Nguyen, Stefan Naulaerts, Alejandra Bruna, Ghita Ghislat, Pedro J. Ballester

**Affiliations:** 1 Cancer Research Center of Marseille, INSERM U1068, F-13009 Marseille, France; nguyencamlinh@hus.edu.vn; 2 Institut Paoli-Calmettes, F-13009 Marseille, France; 3 Aix-Marseille Université UM105, F-13009 Marseille, France; 4 CNRS UMR7258, F-13009 Marseille, France; 5 Department of Life Sciences, University of Science and Technology of Hanoi, Vietnam Academy of Science and Technology, Hanoi 100803, Vietnam; 6 Ludwig Institute for Cancer Research, 1200 Brussels, Belgium; stefan.naulaerts@bru.licr.org; 7 Duve Institute, UCLouvain, 1200 Brussels, Belgium; 8 The Institute of Cancer Research, London SM2 5NG, UK; Alejandra.bruna@icr.ac.uk; 9 Centre d’Immunologie de Marseille-Luminy, INSERM U1104, CNRS UMR7280, F-13009 Marseille, France; gghislat1@gmail.com

**Keywords:** biomarker discovery, machine learning, patient-derived xenograft, precision oncology, tumour profiling

## Abstract

(1) Background: Inter-tumour heterogeneity is one of cancer’s most fundamental features. Patient stratification based on drug response prediction is hence needed for effective anti-cancer therapy. However, single-gene markers of response are rare and/or may fail to achieve a significant impact in the clinic. Machine Learning (ML) is emerging as a particularly promising complementary approach to precision oncology. (2) Methods: Here we leverage comprehensive Patient-Derived Xenograft (PDX) pharmacogenomic data sets with dimensionality-reducing ML algorithms with this purpose. (3) Results: Combining multiple gene alterations via ML leads to better discrimination between sensitive and resistant PDXs in 19 of the 26 analysed cases. Highly predictive ML models employing concise gene lists were found for three cases: paclitaxel (breast cancer), binimetinib (breast cancer) and cetuximab (colorectal cancer). Interestingly, each of these multi-gene ML models identifies some treatment-responsive PDXs not harbouring the best actionable mutation for that case. Thus, ML multi-gene predictors generally have much fewer false negatives than the corresponding single-gene marker. (4) Conclusions: As PDXs often recapitulate clinical outcomes, these results suggest that many more patients could benefit from precision oncology if ML algorithms were also applied to existing clinical pharmacogenomics data, especially those algorithms generating classifiers combining data-selected gene alterations.

## 1. Introduction

### 1.1. Single-Gene Markers, the Predominant Approach to Precision Oncology, Are Scarce

It is now well-established that the efficacy of cancer drugs is strongly patient-dependent. Whereas analgesics, such as Cox-2 inhibitors, show efficacy in 80% of patients, on average, only 25% of oncological patients actually respond to cancer drugs [1]. Consequently, there is a great need to find accurate ways to predict which cancer patients will respond to a given anti-cancer treatment. The predominant approach to date has been to identify a specific somatic mutation to act as a single-gene biomarker discriminating between therapy responders and non-responders [2]. Such a predictive biomarker is commonly referred to as an actionable mutation (either a point mutation, deletion or amplification of a specific gene in the tumour sample). Despite being able to predict the response to some drugs [3,4], most patients cannot benefit from single-gene markers because these have simply not been found for the vast majority of drugs [5,6]. Moreover, drug markers are generally found predictive of a specific cancer type, which means that the marker might not be predictive of drug response on patients from other types [7].

### 1.2. Even FDA-Approved Single-Gene Markers Can Be Weakly Predictive: An Example

**Figure 1:**
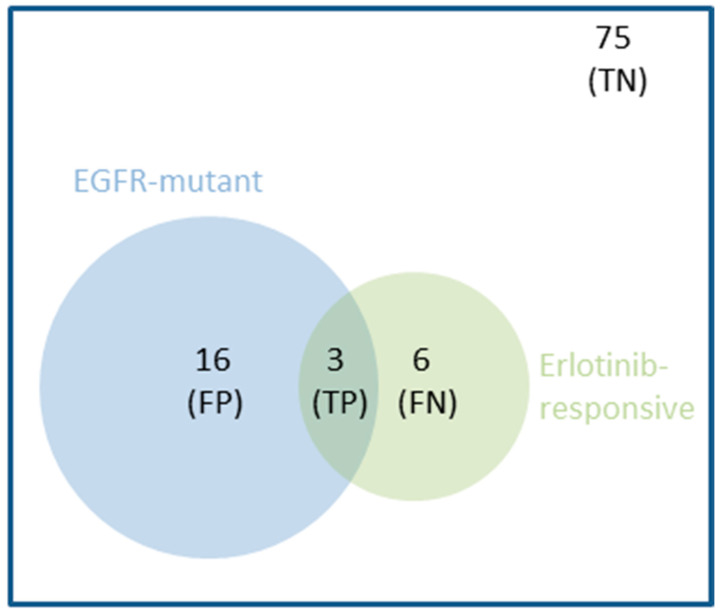
Venn diagram showing the performance of a representative single-gene marker, which predicts that EGFR-mutant NSCLC patients are sensitive to the Erlotinib drug. In a clinical study [8], 100 NSCLC patients were treated with Erlotinib, 19 of them harbouring EGFR-mutant tumours. However, despite being an FDA-approved genomic marker [2], the mutational status of EGFR was a modest predictor of NSCLC tumour response to Erlotinib: 84% (16/19) of EGFR-mutant tumours did not respond. This false positive (FP) rate also means that only 16% (3/19) of these tumours turned out to be responsive (precision of 0.16). Furthermore, 7% (6/81) of NSCLC tumours with wild-type EGFR actually responded to the drug. This false negative (FN) rate means that only 33% (3/9) of the responsive tumours were recalled by this predictor (recall of 0.33). Thus, this case is representative of single-gene markers in that their performance is generally quite modest [5,6]. Intuitively, one can see how combining multiple gene alterations could result in a predictor (blue circle = EGFR-mutant patients predicted to be erlotinib-responsive) having a better overlap with the set of responsive tumours (green circle = observed erlotinib-responsive patients) by decreasing FPs and FNs. The overlap hence corresponds to the number of true positives (TP). Lastly, patients who do not fall into any of these three non-overlapping categories are true negatives (TN).

Not only are these simple drug-gene associations rare, but they are also not strong predictors of drug response in some cases. For example, the mutational status of EGFR in Non-Small Cell Lung Cancer (NSCLC) is an FDA-approved marker of response to Erlotinib [2,8]. The response rate in EGFR-mutant NSCLC tumours was found to be only 16% in this study [8], i.e., a 16% precision. Low precision may be due to the interplay of a range of confounding factors, acting on either the same gene (e.g., low expression of mutant EGFR) or other genes (e.g., resistance-inducing mutations in the TP53 gene [9]). The same study unveiled that 67% of the responsive patients were not correctly identified as such, which corresponds to a 33% recall due to their NSCLC tumours not being EGFR-mutant. This means that two-thirds of NSCLC patients responded to Erlotinib by molecular mechanisms that do not involve EGFR mutations. The Matthews Correlation Coefficient (MCC) summarises both types of error (false positives and false negatives, whose numbers are inversely proportional to precision and recall, respectively) into a single performance metric. This single-gene marker obtained an MCC of just 0.11, which is slightly better than random classification (MCC = 0) and very far from perfect classification (MCC = 1). Figure 1 clarifies the limitations of this single-gene marker further.

### 1.3. Multi-Gene Models Built with Machine Learning Complement Single-Gene Markers

While precision and recall vary depending on the drug and its actionable mutation, the example in Figure 1 is representative in that the values of these metrics are generally quite modest [5,6,10]. Furthermore, it is widely believed that a suitable cancer treatment can only be predicted for those patients who have one of such actionable mutations [5]. We argue, however, that single-gene markers of drug response constitute only one possible approach to precision oncology. Consequently, more patients could benefit from taking an alternative approach that instead captures the interplay between multiple gene alterations that co-operatively control treatment response within tumours of a specific cancer type.

A promising complement to single-gene markers is indeed the application of Machine Learning (ML) [11] to learn which combinations of gene alterations are most predictive of in vivo tumour response to a given treatment. ML algorithms can build in silico models with higher precision (i.e., fewer false positives) by learning which gene alterations, other than the single actionable mutation, influence drug response and how. Regarding increasing recall (i.e., fewer false negatives), ML can potentially learn all the different ways in which tumours of a given cancer type respond to a specific anti-cancer therapy. In that case, ML models would correctly identify not only responders with the actionable mutation as the single-gene marker but also the responders that are wild-type for that gene. Moreover, ML can provide predictive multi-gene models for some of the many drugs for which a single somatic mutation is simply not enough to predict tumour response [12,13].

### 1.4. Learning from Preclinical Data Represents an Alternative When Clinical Data Is Unavailable

Unfortunately, a major limiting factor for the application of ML to this problem is the availability of relevant data [14] (other limiting factors include model selection, bias and overfitting). Although the public release of new clinical pharmacogenomics data sets to power precision oncology is often promised, drug response data is typically excluded from these sets or at the very least limited to a few drug treatments. For example, the first release of the AACR Project Genie [15] contained 19,000 molecularly profiled tumour samples from various cancer types. However, the responses of the corresponding cancer patients to the administered treatments are still withheld to this date. Even if that information was revealed, cancer patients usually receive drugs in combination and/or several lines of therapy after sample collection, which constitute confounding factors in the discovery of new predictors of drug response. Deep molecular profiling, with no treatment response data, is only part of the puzzle.

In the last nine years, data comprising thousands of molecularly-profiled cell lines treated with hundreds of cancer drugs have been made freely available, e.g., GDSC [16], CCLE [17] or CTRP [18]. Cell lines are relatively cheap, quick to grow and amenable to high-throughput experiments [19]. In addition, some cell lines have been shown to mimic primary tumours sufficiently well [19,20,21]. Given their suitability for high-throughput experiments [19], they are also the model for which more data is publicly available [22]. Thus, such in vitro pharmacogenomics data sets are the only ones available to predict response on many drug-cancer type pairs. Despite these advantages, cell lines suffer from several inherent limitations. For example, intra-tumour heterogeneity and extracellular environment are not captured by cell lines, and they are furthermore prone to divergence across passages [20].

In this context, Patient-Derived Xenograft (PDX) models are important when no relevant clinical data is available [23,24,25,26]. PDXs have been reported to capture patient-to-patient response variability to anti-cancer therapy [27,28]. In addition, these preclinical tools preserve, to some extent, the intra- and inter-tumoral heterogeneity observed in the originating cancer sample and the clinical population, respectively [29,30]. Taken together, these results support the use of these PDX models in guiding clinical therapeutic decisions for a more effective cancer treatment management [31,32]. PDX pharmacogenomics data represents an attractive opportunity to build ML models to predict tumour response in those treatment-cancer type pairs for which clinical data sets are not available. Prominent among such data sets stands the NIBR-PDXE [32] resource for its high number of PDXs and their comprehensive profiling. Over 1000 PDXs were established, with 40% of these molecularly profiled at three levels: whole-exome single-nucleotide variants (SNVs), copy-number alterations (CNAs) and gene expression (GEX). Importantly, some of these PDXs were also evaluated with a panel of 60 treatments, which makes these data sets amenable to ML modelling.

### 1.5. A Comparison of Multi-Gene vs. Single-Gene Models across In Vivo Preclinical Models Is Lacking

Here we carry out a systematic study to investigate how ML can improve the prediction of in vivo drug response from tumour molecular profiles by combining multiple gene alterations. To the best of our knowledge, we were the first to analyse NIBR-PDXE data with this aim. Note that previous studies applying ML to this problem across multiple drugs have been based on pharmacogenomics data from in vitro cell lines, instead of PDXs. Three additional aspects of this paper are novel: (1) directly comparing the performance of ML classifiers against that of single-gene markers across multiple treatments, (2) introducing and applying a variant of ML classifier intended to provide a more stringent Feature Selection (FS) [33] as a way to better handle the high-dimensionality of data sets and (3) adopting a non-competitive approach where the goal is to identify the most suitable profile and classifier type for each treatment. This is in sharp contrast with the current one-size-fits-all approach, where only the model with the highest average performance across treatments is selected [34].

Given that single-gene markers based on somatic mutation data are central to current genomic medicine [6,35], it is surprising that practically all studies evaluating multi-gene ML models have not included a direct comparison with single-gene markers. When we recently carried out such a comparison in vitro across 127 drugs [13], we observed that ML models combining multiple gene alterations identified a higher proportion of drug-sensitive cell lines, i.e., had a higher recall, in 93% of the drugs. Here we will determine whether this is also the case in vivo. If confirmed in vivo, this would mean that many more patients could benefit from precision oncology if multi-gene ML methods are also applied to existing clinical pharmacogenomics data.

One major challenge to build any predictive model is the high dimensionality of pharmacogenomics data. While typically, only tens of tumours have their response to the drug available, the molecular profiles of these tumours may easily aggregate over 50,000 features. To face this challenge, ML algorithms with built-in FS, such as Elastic Nets [36,37,38,39,40], Ridge [37,40], LASSO [37,38,40,41], Random Forest (RF) [13,37,39,40,42,43] or XGBoost [44,45], have been used to model pharmacogenomics data from in vitro cell lines. However, these methods have also been found to be unable to provide predictive models for many drugs. Here we will employ a new strategy, Optimal Model Complexity (OMC), to complement the ability of RF to reduce the dimensionality of tumour molecular profiles. With OMC, only a very small proportion of the typically thousands of features in the considered molecular profile will be employed by the resulting model. This is beneficial in that much fewer features would have to be experimentally determined in forthcoming tumours. In addition, in those cases where a few tumour features control drug response, predictions should tend to be more accurate because RF will no longer be considering the thousands of irrelevant features that promote model overfitting.

Lastly, the application of an ML method achieving slightly better performance than a previous method on average across drugs is commonly reported. However, ML methods can perform similarly on average over a set of drugs but dissimilarly on one of those drugs. Therefore, we adopt a non-competitive approach instead, where the goal is to identify the most suitable profile and classifier type for each treatment. This approach should be collectively more predictive than using the method with the highest average performance across treatments. Another expected advantage is that we will be able to find out which molecular profile is most predictive for a given drug, which is valuable to reduce the time and cost that would be associated with determining the rest of the profiles on patients treated with that drug.

## 2. Materials and Methods

### 2.1. NIBR-PDXE Data

The NIBR-PDXE data set [32] is publicly available at https://static-content.springer.com/esm/art%3A10.1038%2Fnm.3954/MediaObjects/41591_2015_BFnm3954_MOESM10_ESM.xlsx (accessed on 25 September 2021). This Excel file has five tabs named *RNASeq_fpkm*, *copy_number*, *pdxe_mut_and_cn2*, *PCT_raw_data* and *PCT_curve_metrics*. The first three tabs contain three molecular profiles of the xenografted tumours. The *RNASeq_fpkm* tab contains gene expression values. The copy number tab contains the actual copy number of each gene. Copy number is also available as a categorical variable at the *pdxe_mut_and_cn2* tab (this table also contains detected mutations per gene). Around 400 PDX models were profiled at each of these omic levels. The other two tabs are for treatment response data. The raw response data tab (*PCT_raw_data*) includes the percentage of tumour volume change relative to tumour volume at the start of treatment (%∆TVol) of each treated PDX recorded every 3–4 days. Lastly, the processed response data tab (*PCT_curve_metrics*) includes the categorisation of PDX responses into one of four classes calculated from raw response data. Further information about how this data set was generated can be found in the original study [32].

### 2.2. Processing Treatment Response Data for Modelling

For each treated PDX, we retrieved its category from the processed response data. We also calculated its category from raw response data as indicated by Gao et al. [32]. Retrieved and calculated response categories differ in 277 of the 4758 PDX-treatment pairs. Although such discrepancies were small (mostly swaps between contiguous categories) and not numerous (5.8% of the cases), we decided to use the calculated categories in these cases so that all PDX-treatment pairs were categorised following the same set of rules (Figure S1). Gao et al. [32] further subdivided PDX response into two classes: responders, as those PDXs exhibited some level of sensitivity to the treatment (CR, PR or SD), and non-responders (PD), as those resistant to the treatment.

### 2.3. Processing Molecular Profiling Data for Modelling

For each gene, the Single-Nucleotide Variant (SNV) feature for that gene is assigned a value of one if at least one SNV was detected in this gene region (i.e., reported in the *pdxe_mut_and_cn2* tab). If no SNV was detected in this gene, the gene was labelled as Wild Type (WT), and the SNV feature was assigned a value of zero. This encoding scheme is commonly used [46,47,48], as it has the advantage of leading to a much less sparse data instances vs. features matrix than if a binary feature was defined for each SNV (a gene typically has several SNVs). In this way, each PDX profiled at the SNV level was characterised by a set of 15,232 binary features (one feature per gene). On the other hand, the *copy_number* tab contains the actual copy number (CN) of each gene as determined by Gao et al. [32]. This is the CN profile composed of 23,853 real-valued features (one per gene) and was not analysed by Gao et al. Instead, these authors categorised these measurements as follows: Amp5 if the gene is moderately amplified with copy number in the range ≥5 and <8, Amp8 if the gene is strongly amplified with copy number ≥8 and Del0.8 if the gene is deleted with copy number ≤0.8 (these per-gene CN categories were reported in *pdxe_mut_and_cn2*). As previously performed in [46,47], we binarised copy-number data to generate less sparse features: the Copy-Number Alteration (CNA) feature of a gene has a value of one for aberrant copy number (Amp8, Amp5 or Del0.8) and zero otherwise. Thus, each PDX profiled at the CNA level is described by a set of 21,534 binary features (one per gene). By contrast, the fourth molecular profile, Gene Expression (GEX), is directly the data provided in the *RNASeq_fpkm* Table. Thus, each PDX profiled at the GEX level is characterised by 22,665 real-valued features (one per gene as well).

### 2.4. Processed Data Sets for Modelling

Only a part of the PDX models from NIBR-PDXE have been both treatment-response and molecularly profiled. A previous cancer pharmacogenomics modelling study showed that it is possible to predict the drug response of held-out tumours with an ML model trained on just 35 tumours [49]. As we are not aware of successful studies using smaller training sets, we focused on the two cancer types with the highest numbers of profiled PDXs per treatment, Breast Cancer (BRCA) and Colorectal Cancer (CRC), where all but one of these 26 treatment-cancer type pairs had at least 35 PDXs. As we are using CV [50], each PDX appears exactly once in the test set. Employing nested CV on algorithms requiring model selection, here, those employing OMC, provides an unbiased estimate of model performance, as it has been shown in [51,52]. Thus, the CV performance of a model is always based on the prediction of test PDXs that were not used in any way to train or select the model (i.e., any feature selection is hence carried out on training folds only). With these settings, a set of 13 treatments were administered to BRCA PDX models, and another set of 13 treatments were administered to CRC PDX models. The first two tabs of the NIBR-PDXE data (available at https://static-content.springer.com/esm/art%3A10.1038%2Fnm.3954/MediaObjects/41591_2015_BFnm3954_MOESM10_ESM.xlsx (accessed on 25 September 2021)) state the numbers of sensitive and resistant PDXs per treatment for BRCA and CRC, respectively.

Note that no claim about the optimality of the 35PDXs threshold is made. Although less likely, some cases with fewer PDXs might be predictive too. In any case, a threshold is required to make the analysis manageable. We run 2 ML algorithms for 2 cancer types (BRCA and CRC), 13 treatments per cancer type, 4 types of molecular profiles for each treatment-cancer type pair and 10 replicates. Thus, a total of 2080 Leave-One-Out (LOO) Cross-Validations (CV) runs have been carried out. A less restrictive threshold would further increase the number of trained and tested ML models, as well as the subsequent analysis, which is already extensive for a single study.

### 2.5. Measuring the Predictive Performance of a Classifier

The pharmacogenomics data set for a given cancer type, molecular profile, and the *i*th treatment can be represented as:

(1) $$D_{i}={\left\{\left(class_{i}^{\left(k\right)},x_{}^{\left(k\right)}\right)\right\}}_{k=1}^{k=n_{i}}$$

where x is a high-dimensional vector with the features from the considered profile and the *i*th treatment has been administered to *n_i_* PDX models. In these binary classification problems, positive data instances are PDXs sensitive to the considered treatment (class = sensitive), whereas negatives are resistant PDXs (class = resistant). Note that, while they have slightly different meanings, we use the terms responder and sensitive PDX interchangeably as it is customary (same applies to the terms non-responder and resistant PDX).

Each of these data sets is employed to train classifiers to predict the class of a PDX from its corresponding molecular profile. Classifiers not employing model selection (i.e., RF with fixed hyperparameters) are evaluated with standard LOOCV, whereas those employing model selections are evaluated with nested LOOCV to avoid overestimating their performance [51,52]. It is worth noting that nested LOOCV is nothing but a standard LOOCV where the model optimised (selected) with the training set of a given fold is applied to the test set of that fold (i.e., instead of training and testing the same model per fold). In other words, the inner LOOCV is here used to select the model with the optimal complexity to predict its outer left-out data instance (PDX in this context), then all predictions from the outer LOOCV are used to calculate the reported MCC value.

Once observed classes are compared to predicted classes, PDXs in the considered data set can be broken down into true positives, true negatives, false positives and false negatives (their numbers being TP, TN, FP and FN, respectively). Thus, the discrimination offered by a classifier can be summarised by the Matthews Correlation Coefficient (MCC) [53].

(2) $$\mathrm{MCC}=\frac{\mathrm{TP}\cdot \mathrm{TN}-\mathrm{FP}\cdot \mathrm{FN}}{\sqrt{\left(\mathrm{TP}+\mathrm{FN}\right)\cdot \left(\mathrm{FN}+\mathrm{TN}\right)\cdot \left(\mathrm{TN}+\mathrm{FP}\right)\cdot \left(\mathrm{FP}+\mathrm{TP}\right)}}$$

MCC can take values from −1 to 1, where 1 means that the classifier provides a perfect agreement between the observed and predicted classes, −1 indicates a perfect disagreement and 0 means that the classifier performance is equivalent to that of predicting the class at random.

To investigate how the two sources of error contribute to the overall predictive performance represented by MCC, we also calculate Precision (PR) and Recall (RC) for each predictor. PR and RC are two classical metrics [54] whose definitions are:

(3) $$\mathrm{PR}=\frac{\mathrm{TP}}{\mathrm{TP}+\mathrm{FP}}\;\mathrm{RC}=\frac{\mathrm{TP}}{\mathrm{TP}+\mathrm{FN}}$$

In this study, a PR value of 0 would mean that all the PDXs predicted sensitive by the classifier are actually resistant, whereas a PR value of 1 would indicate that all the PDXs predicted responsive were experimentally confirmed to be responsive. On the other hand, RC is 0 when none of the sensitive PDXs is correctly identified as such, whereas RC is 1 if no sensitive PDX is missed by the classifier. An alternative global metric capturing both types of errors is the F-score (F1), which is the equally-weighted harmonic mean of PR and RC. The optimal value of F1 is 1 (when both PR and RC are maximal or 1), whereas its worst value is 0 (when either PR or RC is 0; when both PR and RC are 0, F1 is also 0).

The Receiver Operating Characteristic (ROC) curve and the Area Under the Curve (AUC) were calculated using the R package ROCR version 1.0–7. The ROC curve was built based on the response class predicted by the model (RF-OMC or SG) and the actual observed class of each test PDX. For a given model, the ROC curve shows all different pairs of values for the metrics True Positive Rate (TPR) and False Positive Rate (FPR) resulting from varying the operating threshold from 0 to 1 to cut-off the probability of the PDX to be a positive (responsive). RF-based models assign each test PDX with a class probability value between 0 and 1; thus, their ROC curves have one point for each threshold leading to a different TPR-FPR pair. By contrast, SG markers predict the class of each test PDX as either resistant/non-responsive (0) or sensitive/responsive (1); thus, all thresholds between 0 and 1 lead to the same TPR-FPR pair (i.e., a single intermediate point in the ROC curve apart from the two extreme values). The AUC is the area under the ROC curve. AUC ranges from 0 to 1. AUC = 1 denotes a perfect classification, whereas AUC = 0.5 corresponds to a random-level prediction of classes.

### 2.6. Multi-Gene Classifiers with Built-In Feature Selection (FS)

Some ML algorithms can construct classifiers with built-in FS to mitigate the impact of the high dimensionality of data on their generalisation to unseen data. Random Forest (RF) [55] is one of these algorithms, as it generates trees that ignore irrelevant features by construction and thus is often found effective in modelling high-dimensional omics data [56]. We used the following values for RF hyperparameters: 1000 for the number of trees (a high number is recommended [57], and further reduction of error was not observed with RF using more than 500 trees [58]) and the square root of the number of considered features for m_try_ (this a default value that has been found to work well across binary classification problems [58]). We preferred this to tune these hyperparameters for each training set, as RF tuning generally results in very small improvements at the cost of being much more computationally expensive [42,58,59,60]. As no model selection was carried out for this algorithm, standard LOOCV was performed to estimate the performance of RF using all the features (RF-all) on each data set (treatment-cancer type-molecular profile). To assess the variability introduced by the stochastic character of RF, we perform 10 repetitions of LOOCV per case, each using a different random seed (see corresponding boxplots in Figures S4 and S5).

The proportion of responsive and non-responsive PDXs changes from case to case (see tabs *data_by_treatment_BRCA* and *data_by_treatment_CRC* in the Supplementary Tables). Although class imbalances are not strong, these could still introduce some loss in performance. Thus, we enabled class weighting in the RF algorithm (R package ‘randomForest’ version 4.6–12), which counterbalances class imbalances by putting a heavier penalty on misclassifying the minority class. The misclassification penalty of the minority class was set to the proportion of the majority class, thus promoting RF trees that are equally accurate regardless of the class.

### 2.7. Multi-Gene Classifiers with Optimal Model Complexity (OMC)

An effective way to improve predictive performance is to reduce the dimensionality of the data. Here data dimensionality can be defined as the number of considered features over the number of PDXs. One route to reduce dimensionality is hence to use more training data, but these are usually not available. An alternative route is to only consider the most informative features in the data, thus typically discarding the many thousands of less informative features (hence strongly reducing data dimensionality while retaining most of the initial information content).

However, the optimal number of features and their identities depend on various factors (treatment, profile, cancer type and data set). Consequently, we designed OMC as a strategy to build ML models, employing only the most relevant features. In a nutshell, OMC is made of three modules: one to rank features according to their relevance to treatment response, another to train an ML model per considered subset of features and a third one to select the optimal model among those trained. Regarding ranking features, we used *p*-values from two-sided Fisher’s exact tests to rank binary features within a given binary profile (SNV or CNA) and *p*-values from two-sided unpaired *t*-tests to rank real-valued features (GEX or CN). Each *p*-value measures how strongly that feature discriminates between responsive and non-responsive PDXs. For each profile, treatment and cancer type, we considered n/2 subsets of features (n is the number of PDXs available for that case): the subset with the top 2 features, that with the top 3 features, …, that with top n/2 features and finally all features for that profile. This limit of n/2 ensures that the ML algorithm will not be challenged by high-dimensional data (i.e., all trained models will have at least two data points per considered feature), except for the run using all features intended to find out whether the case requires more than the top n/2 features. Lastly, the best among these n/2 models is selected as that with the highest LOOCV MCC. To estimate its performance, nested LOOCV MCC is calculated with model selection in the inner loop [51,52] (the same values of the hyperparameters used for RF-all were also used here for RF-OMC). Sometimes the best RF-OMC model is that only employing the top 2 or the top 3 features (i.e., more complex models are not more predictive). As the RF model reduces in these cases to a set of redundant shallow trees, we are probably wasting computer time, as running RF with 10–50 trees should do just as well.

### 2.8. Random Model Based on the Prior Probability of Each Case

The following protocol was followed for each case. First, the proportion of sensitive PDXs in each LOOCV training fold was taken as an estimate of the probability of a PDX being sensitive. Second, a random number between 0 and 1 was generated according to the estimated prior probability to decide whether the held out PDX in the LOOCV test fold was predicted as sensitive or not. Third, the process was iterated over all LOOCV folds. Once the response class of every PDX had been predicted in this way, a single MCC value was computed from predicted and actual classes of the PDXs. Lastly, we repeated this process 10 times with the same set of 10 different random seeds employed with RF-OMC and RF-all. From the 10 MCCs for the case and the other 10 MCCs for the RF-model (either RF-OMC or RF-all), we carried out a one-sided paired *t*-test to determine whether the performance of the RF model was better than that of this random model (*p* < 0.05). Results can be found in Figures S2–S5 (each point in a boxplot there represents the MCC of one of the 10 LOOCV runs).

### 2.9. Single-Gene Markers

We identified the best single-gene marker for each of the 26 treatment-cancer type pairs using exactly the same data as RF-all and RF-OMC via LOOCV. The SNV profile was used as the source of detected somatic mutations, as there is currently a strong interest in using them as pharmacogenomic markers in oncology [5]. For each fold, the response of the PDX held-out in the test fold was predicted with the most significant sensitive marker (i.e., predicted sensitive if an SNV is detected in the marker gene, predicted resistant otherwise). Such marker was determined by calculating two-sided Fisher’s exact tests across training fold PDXs, one per gene, leading to *p*-values and effect sizes (ϕ [61]) for 15,232 genes. The gene with the lowest *p*-value among those constituting sensitive mutations (ϕ > 0) was identified. The operation was repeated for each fold resulting in a LOOCV predicted class for each treated PDX and thus the LOOCV MCC for the best marker. After evaluating its predictive performance, we recalculated each best single-gene marker, now using all the data so that these markers are ready to be used on forthcoming tumours. These results are in the Supplementary Tables (*single-gene_marker* tab).

## 3. Results

We started the analysis by determining which drug-cancer type pairs in NIBR-PDXE [32] have sufficient data to be likely to lead to predictive models (see the Methods section). We identified 13 of such treatments in Breast Cancer (BRCA) and another set of 13 treatments in Colorectal Cancer (CRC). All but one of these 26 treatment-cancer type pairs had at least 35 PDXs, each PDX with treatment-response, SNV, CNA and GEX profiles.

### 3.1. Establishing the Best Multi-Gene Predictor for Each Treatment and Cancer Type Pair

To perform this task, we trained and evaluated two ML algorithms on each data set using LOOCVs, as detailed in the Methods section. The first algorithm is RF using all available features (RF-all), whereas the second is an OMC variant of RF to identify the most predictive features in each case (RF-OMC). Both algorithms are used to build binary classification models. To account for their stochastic nature, we trained each algorithm on each LOOCV training fold 10 times, thus obtaining 10 estimations of each performance metric per case. Note that this study always reports the median performances of each algorithm on held-out PDXs that were not used to train or select the model providing the prediction (e.g., each reported MCC is the median of 10 MCC determinations from 10 independent nested LOOCVs). To assess which cases are better predicted by a particular gene, we also performed the standard single-gene analysis to evaluate which genes sensitise PDXs to the treatment when an actionable SNV is detected. In particular, we identified the sensitive marker with the lowest *p*-value and reported its LOOCV performance. Full results can be found in the “single-gene_markers” tab of the Supplementary Tables.

**Figure 2:**
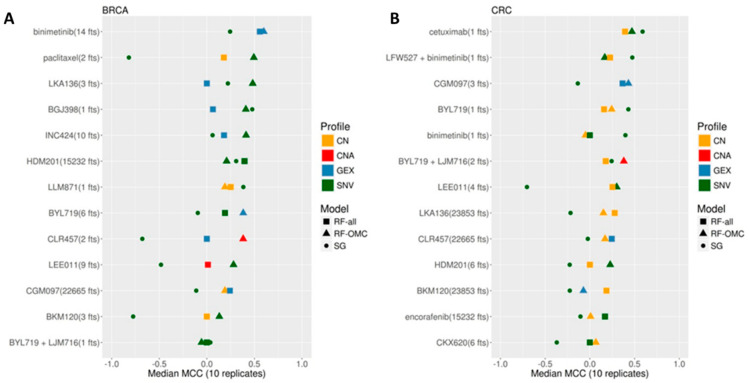
Predictive performance of the best single-gene (SG) marker, Random Forest (RF) with Optimal Model Complexity (RF-OMC) and RF using all features (RF-all). (**A**) Best predictor per treatment and classification model type on BRCA PDXs. Each row shows the results for one treatment and shows the number of top features with which the best classifier for that treatment was trained on. Furthermore, the colour and shape of each classifier indicates the employed molecular profile and model type, respectively (“*fts*” stands for “features”). For instance, paclitaxel (2 fts) appears as a green triangle, which means that paclitaxel had RF-OMC-SNV as the classifier with the largest median MCC on LOOCV held-out PDXs, and this classifier employed the top two features from the SNV profile (MUC20 and UPK3BL). All treatments have at least one predictor performing above random level (MCC = 0), with the accuracy in predicting BRCA PDX response being strongly treatment dependent. Importantly for clinical implementation, the best classifier per treatment is usually a model that only requires a handful of features to operate. (**B**) Best predictor per treatment and classifier type on CRC PDXs. Strong predictors of treatment response were also found for this cancer type. However, there were fewer of these predictive models in CRC than in BRCA (five treatments were predicted with MCC > 0.4 in BRCA, but only two treatments were predicted at this level in CRC). Top models are more frequently associated with CN profiles in CRC than in BRCA. It is also clear that CNA profiles, using CN as a binary feature (altered/wild type), leads to less predictive models than real-valued CN.

Figure 2 shows the validation results for each of the 26 cases. We found that the accuracy in predicting treatment response on left-out PDXs strongly depends on the considered treatment, molecular profile and classifier type in both cancer types. The performances of the best predictors from each case range from being slightly above purely random classification (MCC = 0) to high in the context of this problem (MCC = 0.57). In addition, a large variability is often obtained across the four molecular profiles within the ML algorithm. For each ML algorithm, we show the performance of the most predictive molecular profile for that case. As CNA is just a binarisation of the real-valued copy number (CN) profile, information-richer CN is used much more often than CNA in the best models across cases (19 vs. 3 models in Figure 2, respectively). The performance also varies strongly across the three model types within a given case.

RF-OMC not only leads to more accurate predictors in 13 of the 26 cases, but these predictors merely require a concise subset of all gene alterations to operate (these concise gene lists are reported in tab RF_predictors of the Supplementary Tables). By contrast, solely 6 of the 26 cases were better predicted by RF-all. Figure 2 shows that the MCCs of RF-OMC across the 13 treatments were not better than those of RF-all in CRC (*p* = 0.47 from a one-sided paired *t*-test, both algorithms obtaining an average MCC of 0.19). However, RF-OMC was found to outperform RF-all in BRCA (*p* = 0.009 from a one-sided paired *t*-test, average MCCs of 0.32 and 0.16, respectively). The best predictor in the remaining seven cases, mostly in CRC, was a single-gene marker based on SNV data (a comparison between single-gene and multi-gene classifiers restricted to SNV data can be found in the γ plots in Figures S7 and S8). Overall, these results stress the importance of considering several model types and profiles to predict in vivo treatment response.

For further validation, we have also compared each of these two ML classifiers against a random model where the response class is predicted using the prior probability of a PDX to be sensitive. Figures S2 and S3 show that RF-OMC predicts 21 of the 26 cases better than random (*p* < 0.05; one-sided paired *t*-test). Highly significant *p*-values strongly suggest that the corresponding lists of selected features have a high predictive value. By contrast, Figures S4 and S5 show that RF-all is only able to predict 16 of the 26 cases at this level (*p* < 0.05; one-sided paired *t*-test).

Each of the models in this study is evaluated using independent data by LOOCV (i.e., the measured response of any test PDX was not used in any way to train or select the model). CV is a standard way to assess predictive accuracy not only in this topic [13,62,63,64], but in any problem where there are few data instances (here PDXs) to train, select and test a machine-learning model [65]. Additional experiments using data from a second PDX resource would be, in principle, appealing. However, we are not aware of another resource releasing data that could be used as a second test set for the here developed models (i.e., PDXs with the same profile, drug and cancer type). Furthermore, even if these experiments were possible, inconsistencies arising from the lack of standardisation in such high-throughput efforts would be likely to be present, as has been the case with in vitro pharmaco-omic resources [66]. Therefore, such additional test sets would evaluate not only the predictors but also the impact of differences in the experimental setup generating the data.

In the next three subsections, we describe the three predictors found to have the highest accuracies in more detail. These three are the most useful models to predict which forthcoming PDXs will be responsive. Table 1 summarises the results of these models.

### 3.2. Predicting BRCA PDX Response to Binimetinib

The best multi-gene predictor for BRCA PDXs treated with binimetinib, a MEK1/2 inhibitor, was RF-OMC applied to GEX data comprising the expression values of 22,665 genes. Our analysis discarded the other three molecular profiles for binimetinib-BRCA (SNV, CN and CNA), as they were substantially less predictive than the GEX profile in this case (the performance of all profiles for each of the 26 cases can, however, be found in the Supplementary Tables). RF-OMC practically offered the same performance as RF-all (MCC of 0.57 vs. 0.56, respectively). However, RF-OMC identified 14 out of these 22,665 genes as the more informative to predict BRCA PDX response to binimetinib. The resulting RF-OMC predictions are hence optimal combinations of the expression values of only these 14 genes, whereas RF-all was trained on all 22,665 GEX features.

**Figure 3:**
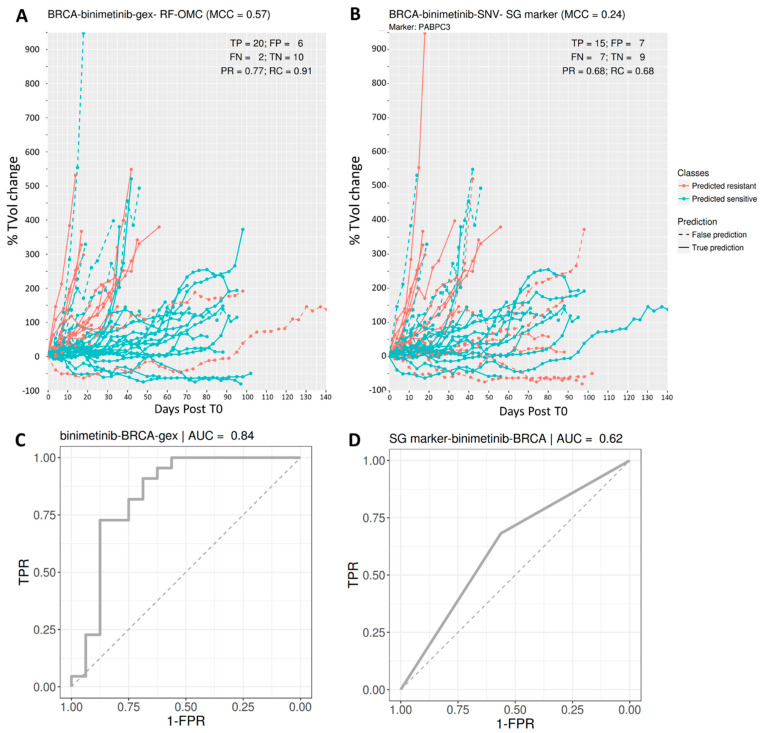
Predicting BRCA PDX response to the MAPK inhibitor binimetinib. (Upper) Visualisation of tumour response prediction for test PDXs. Each line represents one PDX. The vertical axis shows the % of tumour change relative to the tumour volume at time zero (T0; i.e., immediately before the first administration of the drug), and the horizontal axis shows when measurement time in days after T0. From the legend, red discontinuous lines represent false negatives (responsive PDXs that were predicted to be non-responsive), and blue discontinuous lines represent false positives (non-responsive PDXs that were predicted responsive). The better the predictor is, the higher the proportions of lines at the bottom appear in blue (higher recall) and at the top in red (higher precision). (**A**) *Binimetinib (14 fts)*: RF-OMC model predicts BRCA tumour response to binimetinib by optimally combining the expression levels of only 14 genes (CRB3, NDUFA1, MPG, ECI1, ING2, KIF9, TSTD1, FAM100A, TCEAL3, HAGH, PEX11G, SNORA72, SNORA70 and PIN1). A high level of predictive accuracy was achieved on PDXs not used to train the model: MCC = 0.57 (PR = 0.77 and RC = 0.91). (**B**) *Binimetinib (1 fts)*: Using the same input data and evaluation protocol, the best single-gene marker of binimetinib sensitivity was the mutational state of PABPC3. The RF-OMC model obtained a substantially higher level of prediction than that of this standard single-gene procedure: MCC = 0.24 (PR = 0.68 and RC = 0.68). (**C**,**D**) ROC curves and their AUC values to compare the discrimination offered by both models across all possible operating thresholds. The corresponding AUC value was indicated on the top of the ROC curve. This alternative performance metric also assigns higher predictive performance to the multi-gene RF-OMC model.

Figure 3 displays the performance of this multi-gene predictor compared to that of the best single-gene marker for binimetinib-BRCA (the mutational state of PABPC3 with *p* = 0.02 from a two-sided Fisher’s exact test). The multi-gene predictor achieves a more substantial discrimination between sensitive and resistant PDXs than the PABPC3 marker. This is also indicated by a higher MCC (0.57 vs. 0.24). To help to understand what MCC values represent in terms of achieved discrimination, we also indicated FP and FN errors from both classifiers.

### 3.3. Predicting BRCA PDX Response to Paclitaxel

The best multi-gene predictor for BRCA PDXs treated with paclitaxel was RF-OMC applied to somatic mutation data comprising the presence or absence of an SNV in 15,232 genes. Our analysis discarded the other three molecular profiles for paclitaxel-BRCA (GEX, CN and CNA), for being less predictive than the SNV profile in this case (all predictive performances are in the Supplementary Tables). The resulting RF-OMC model employs two out of these 15,232 genes (MUC20 and UPK3BL).

Incidentally, we would like to highlight that feature selection is embedded in RF [67]. RF-OMC generally performs better than RF-all because it promotes a much more stringent feature selection, which is more suitable in most cases. We illustrate this point with paclitaxel-BRCA-SNV as an example. RF-OMC with only two SNV features achieves an MCC of 0.49. By contrast, with all features and the same data, RF-all obtains a far worse MCC of practically zero (MCC = −0.07). Out of these 15,232 features, only 3374 are actually used by any of the 1000 trees forming the RF-all model, as each RF tree only uses those features providing the best discrimination among the m_try_ randomly chosen at each node. The poor performance indicates that 3374 features are still far too many for this particular case, which is much better predicted by an RF model employing the two most predictive features.

**Figure 4:**
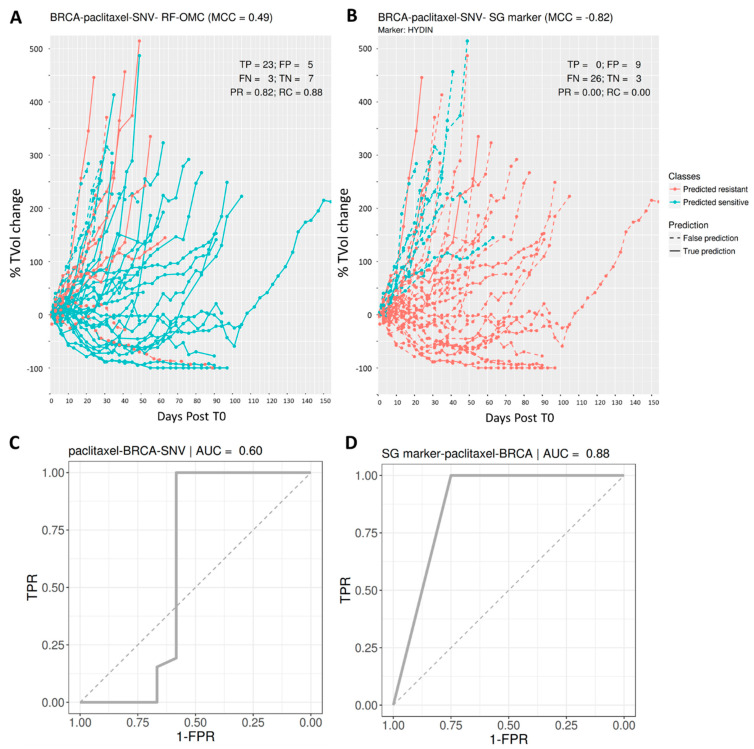
Predicting BRCA PDX response to the tubulin inhibitor paclitaxel. (Upper) Visualisation of tumour response prediction for test PDXs. (**A**) *paclitaxel (2 fts)*: RF-OMC predicts BRCA tumour response to paclitaxel by optimally combining the mutational states of two genes (MUC20 and UPK3BL). A high level of predictive accuracy was achieved on PDXs not used to train the model: MCC = 0.49 (PR = 0.82 and RC = 0.88). (**B**) *paclitaxel (1 fts)*: Using the same input data and evaluation protocol, the best single-gene marker of paclitaxel sensitivity was the mutational state of HYDIN. The RF-OMC model obtained a much higher level of prediction than that of this standard single-gene procedure: MCC = −0.82 (PR = 0.00 and RC = 0.00). (**C**,**D**) ROC curves and their AUC values to compare the discrimination offered by both models across all possible operating thresholds. The corresponding AUC value was indicated on the top of the ROC curve. This alternative performance metric also assigns higher predictive performance to the multi-gene RF-OMC model.

Figure 4 visualises the high performance of this two-gene predictor on paclitaxel-BRCA. The performance of the best single-gene marker, also shown, is very poor. This marker is the mutational state of HYDIN, a gene coding for Protein Phosphatase 1’s Regulatory Subunit 31. While we found this sensitising mutation to be the most strongly associated with the cytotoxic drug paclitaxel in BRCA (*p* = 0.04; two-sided Fisher’s exact test), its performance in left-out PDXs suggests that it is a spurious correlation.

### 3.4. Predicting CRC PDX Response to Cetuximab

The best multi-gene predictor for CRC PDXs treated with cetuximab was RF-OMC applied to somatic mutation data. The other three molecular profiles for cetuximab-CRC (GEX, CN and CNA), were discarded for being less predictive than the SNV profile in this case (all predictive performances are in the Supplementary Tables). We identified four out of these 15,232 genes whose combined mutational states provide better prediction than an RF model using the mutational states of all genes (MCCs of 0.47 and 0.39, respectively).

**Figure 5:**
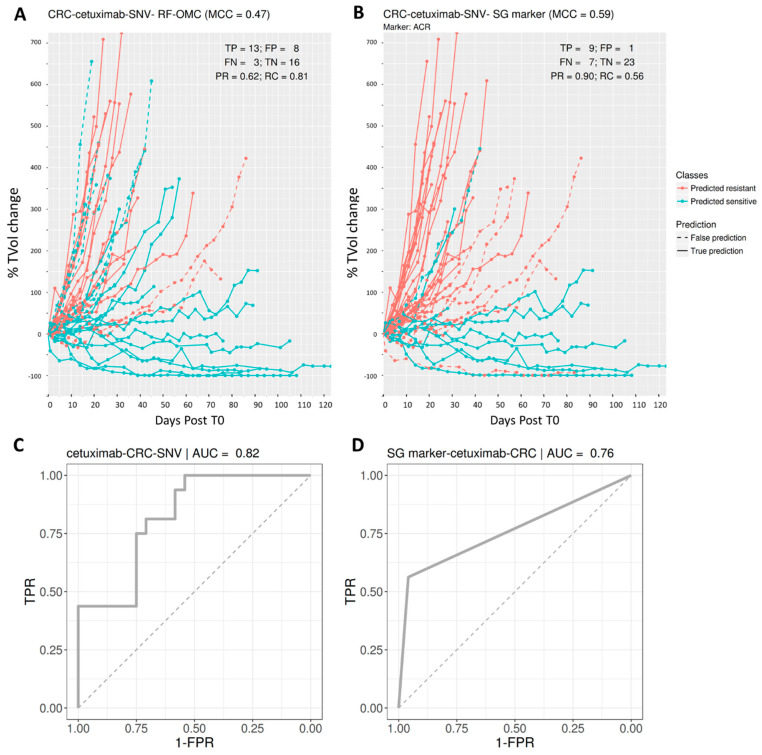
Predicting CRC PDX response to the EGFR inhibitor cetuximab. (**A**,**B**) Visualisation of tumour response prediction for test PDXs. (**A**) *Cetuximab (4 fts)*: RF-OMC predicts CRC tumour response to cetuximab by optimally combining the mutational states of four genes (ACR, DENND4B, NOTCH1 and RPL22). Again, a high level of predictive accuracy was achieved on PDXs not used to train the model: MCC = 0.47 (PR = 0.62 and RC = 0.81). (**B**) *Cetuximab (1 fts)*: Using the same input data and evaluation protocol, the best single-gene marker of cetuximab sensitivity was the mutational state of ACR. The RF-OMC model provided a slightly lower level of prediction than that of this standard single-gene procedure: MCC = 0.59 (PR = 0.90 and RC = 0.56). (**C**,**D**) ROC curves and their AUC values to compare the discrimination offered by both models across all possible operating thresholds. The corresponding AUC value was indicated on the top of the ROC curve. This alternative performance metric now assigns a slightly higher predictive performance to the multi-gene RF-OMC model. As the opposite outcome was obtained with MCC with the default 0.5 threshold, it is expected that optimising this threshold would lead to a more predictive RF-OMC model.

Figure 5 visualises the higher performance of this four-gene predictor on cetuximab-CRC. The performance of the best single-gene marker, also shown, is even higher in this case. This marker is the mutational state of ACR (Acrosin), whose association to this targeted drug is the strongest across the 26 cases (*p* = 0.0003; two-sided Fisher’s exact test). Unlike RF-OMC, most prediction errors correspond to sensitive PDXs that were not correctly identified as such (FN = 7 vs. FP = 1).

### 3.5. Multi-Gene Predictors Generally Offer Substantially Higher Recall Than Single-Gene Markers

**Figure 6:**
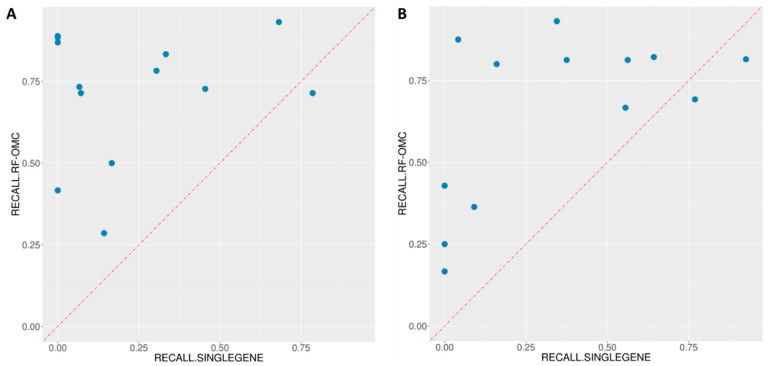
ML multi-gene classifiers exhibit a higher recall than single-gene markers in 23 of the 26 treatment-cancer type pairs. (**A**) In 12 of the 13 treatments for BRCA, multi-gene markers achieve a higher recall than the single-gene marker. The exception was the BGJ398-KIF20B association. (**B**) In 11 of the 13 treatments for CRC, multi-gene markers achieve a higher recall than the single-gene marker. These two single-gene markers with higher recall are the association BYL719-TMEM184A and the association BYL719+LJM716-LSR. Note that these three genes (KIF20B, TMEM184A and LSR) all have very high prevalence (47%, 61% and 85%, respectively) in addition to having the lowest *p*-value as a sensitive marker in their respective cancer types.

In the three cases analysed in Figure 3, Figure 4 and Figure 5, multi-gene predictors exhibit a substantially higher recall than the corresponding best single-gene markers, more concretely, recalls of 0.91, 0.88 and 0.81 (multi-gene) versus recalls of 0.68, 0.00 and 0.56 (single-gene). Figure 6 shows that this is actually a strong general trend: 23 out of 26 studied cases have a higher proportion of correctly predicted sensitive PDXs using the multi-gene markers. We recently observed a similar trend when using the standard version of RF to predict the in vitro drug response from SNV data [13], which is here confirmed in vivo.

In the three cases that do not follow this trend, a slightly higher proportion of correctly predicted sensitive PDXs was found using single-gene markers. The first case is BGJ398-BRCA, and its best single-gene marker is the drug-gene association BGJ398-KIF20B. The other two cases are in CRC and have as best markers the following drug-gene associations: BYL719-TMEM184A and BYL719+LJM716-LSR.

### 3.6. Merging All the Profiles in a Single Set of Features Can Result in a Much Worse Prediction

To shed light on this question, we identified the treatment-cancer type pair whose RF-OMC model obtains the median LOOCV MCC across the 26 pairs, which is BYL719—CRC—CN with an MCC of 0.24. Instead of only using CN features, we merged all the profiles and ran RF-OMC again on this much larger set of features (everything else being equal). The MCC of this model was 0.04, and hence performance degraded in this case by adding many more features.

### 3.7. OMC-Selected Features Are Much More Predictive Than Randomly Selected Features

For each treatment-cancer type pair, we identified the profile with the best RF-OMC model and the number of top features selected by OMC. Then we selected at random from this profile the same number of features. Lastly, the LOOCV of an RF model using these randomly selected features was carried out 10 times, each time with a different random seed. Table 2 shows the results of this experiment. Note that a NA value indicates that the model was not able to LOOCV-predict any sensitive (or resistant) PDXs in at least one of 10 replicates (i.e., either TP + FP = 0 or TN + FN = 0, and hence an MCC NA value). RF models with randomly selected features in Table 2 obtain a worse MCC than the corresponding RF-OMC models in Figure 2 (*p*-value = 0.003, two-sided paired *t*-test).

### 3.8. RF-OMC Can Be Implemented with Alternative Ways of Ranking Features

In RF-OMC, a *p*-value is calculated for each feature according to how well the feature individually discriminates between the two classes (sensitive and resistant). Then features are ranked from the most discriminative to the least discriminative using these *p*-values. Critical features are those that are found to be the most discriminative, and the OMC simply decides the cut-off (the smallest subset of features keeping the largest information content). We could have selected another way to rank features according to how discriminative or predictive they are. For example, one where the correlations between features is taken into account. This is the case of the feature importance tool of RF, where each feature is noised up to measure the resulting decrease in accuracy of the RF model. Thus, the correlation of the noised-up feature with other features influences the ranking of the features by how predictive they are, at the cost of having to run RF with all features to estimate their importance. While investigating this additional algorithm is out of scope, we have illustrated this point in the case of one of the 102 datasets (BRCA- binimetinib, whose most predictive model has the expressions of 14 genes as features, as shown in Figure 3). Figure S10 shows the ranking of these 14 features by *p*-values is similar to that of using RF’s feature importance. The exception is FAM100A, which is much higher ranked when considering its correlation with the other 13 predictive features.

## 4. Discussion

### 4.1. Advantages of Taking a Machine Learning Approach to This Problem

Single-gene markers, such as actionable somatic mutations, are not generally strong predictors of treatment response [5,6,10,12,13]. This situation fuels the need to complement single-gene markers with multi-gene models, especially those built by dimensionality-reducing ML algorithms, which has led to several insights.

First, we have discovered that multi-gene RF models generally retrieve a much higher proportion of in vivo treatment-responsive PDXs than the best single-gene marker for the considered case (see Figure 6). This means that many responders without the marker are now correctly identified as such, owing to an effective combination of multiple gene alterations. In other words, multi-gene predictors of in vivo drug response generally have a higher recall than single-gene markers, which confirms previous findings in vitro [13,43,45]. The recall of a single-gene marker will be necessarily poor in all cases in which the prevalence of the mutation is much lower than the response rate. It is, therefore, not surprising that the three cases where markers have higher recall than RF-OMC in Figure 6 are based on frequently-mutated genes (KIF20B, TMEM184A and LSR contain at least one SNV in 47%, 61% and 85% of the tumours, respectively). Albeit exceptions, this general trend makes sense because a marker by construction can only detect those responsive tumours with the actionable mutation. That is, the marker is blind to responsive tumours arising from alternative molecular mechanisms, as illustrated by the example in Figure 1. By contrast, an ML algorithm can implicitly learn all the mechanisms captured by the training data.

Furthermore, substantially better prediction is achieved if a new variant of RF with enhanced feature selection (RF-OMC) is employed instead of the standard RF algorithm (see Figure S6). An important advantage of RF-OMC over standard RF models is that only a few genes need to be profiled to predict whether a PDX is responsive or not. Concise gene lists in a highly predictive model are valuable for interpretation and clinical application purposes. Take, for instance, the 14 genes forming part of the binimetinib-BRCA GEX predictor (Figure 3). RF-OMC unveiled that this gene list is a promising starting point for mechanistic studies. Such studies would aim at explaining how the nonlinear interplay between the expression values of these genes accurately predicts BRCA PDX response to binimetinib. On the other hand, concise gene lists permit cheaper and faster clinical implementation. For example, instead of carrying out three whole-exome molecular profiles per tumour sample, we now know that it suffices to determine the mutational status of just two genes to predict BRCA tumour response to paclitaxel (Figure 4). This example also highlights that the most responsive tumours to a cytotoxic drug can also be accurately predicted. The latter indicates that the applicability of precision medicine in the current standard of care oncological therapeutic regimes is not restricted to targeted agents but also includes cytotoxic chemotherapy [13,68]. While this is not the aim of the study, we have commented on the cancer relevance of the genes selected to predict treatment response for the three best RF-OMC predictors. This literature review can be found in the supplementary information file (pages 7–14).

In terms of performance, the most predictive multi-gene ML model achieved an MCC of 0.57 (all MCCs were calculated on left-out PDXs that were not used in any way to train or select the model providing that prediction). We are not aware of similar studies using PDX data, but there are a number of them exploiting cell line data. Thus, it is possible to put this level of predictive accuracy in the context of other test set performances reported in the literature for this problem. One study [69] applied several ML algorithms to predict pancancer cell line response from transcriptomic profiles, obtaining MCCs below 0.6 in all cases (see Figure 1 in that paper). Maximum MCCs slightly above 0.5 and 0.3 were also obtained using RF with transcriptomic profiles [43] and genomic profiles [13], respectively. Another study [70] also predicted drug response using many hundreds of pancancer cell lines and several ML algorithms from various omics profiles (gene expression, copy-number alterations, single-nucleotide mutations). Average MCCs across drugs and profiles range from 0.15 to 0.31 or from 0.22 to 0.45 (see Tables 1 and 2 in [70]) depending on the considered data resource. Yet another example is [71], where ML models using the same subset of transcriptomic features for all drugs and ML models using a subset of transcriptomic features specific to each drug reached maximum MCC values of 0.27 and 0.25, respectively. Furthermore, this study [72] used gene variants as features, with MCCs range across drugs from 0.32 to 0.56 or from 0.30 to 0.44 depending on data resource (see Tables 1 and 2 in that paper). Lastly, single-gene drug response markers identified by MANOVA and Chi-Square tests on pancancer cell lines obtained maximum MCCs of 0.30 and 0.31, respectively [48].

How was this level of performance achieved? Considering multiple types of classifiers and molecular profiles collectively improved the prediction of in vivo tumour response across treatments and cancer types. We observed that combining multiple gene alterations via ML results in better discrimination between sensitive and resistant PDXs in 19 of the 26 analysed cases. More importantly, this ML approach has determined the most predictive classifier type and molecular profile for each treatment—cancer type pair. Despite training on data from practically the same PDXs within a cancer type, we found that some treatments can be predicted much better than others (this is true for both cancer types). The results show that increasing the number of considered classifiers leads to higher average accuracy across treatments (see plots α to γ in Figures S7 and S8 with up to three classifiers). Therefore, an effective way to improve the prediction of a given case is to evaluate several model types. For instance, the collective prediction of these 26 cases would have been much worse if we had only considered the standard RF algorithm (see, for instance, the difference between the MCCs of square and triangle signs for drug LKA136 in Figure 2).

Another effective way to improve the prediction of a given case is to consider all the available molecular profiles. It is noteworthy that the results would have also been worse if only one profile had been employed. For instance, 21 of the 26 cases were better predicted by profiles other than GEX (see MCCs of blue signs in Figure 2). This new knowledge is displayed in Figure 2 and fully reported in the Supplementary Tables. By contrast, we did not focus on the integration of all the profiles because this would increase time and cost expenses for each patient in a clinical setting. A secondary reason is that integrating multiple omics profiles may provide no benefit, despite the higher information content, due to the harder challenge of tackling even higher-dimensional problems. Here we presented an example showing that this might even lead to a large performance decrease. In practice, when a multi-omics integration has been reported to result in a benefit, this has typically been incremental [49].

### 4.2. Limitations and Recommendations to Mitigate Them

We have seen that even ML algorithms with built-in FS can often struggle to provide predictive classification models. This is due to overfitting caused by training on high-dimensional data [73,74], and we have introduced and evaluated RF-OMC to mitigate this issue. OMC ranks features by their individual ability to discriminate between resistant and sensitive tumours (e.g., via a *t*-test), with only the highest-ranked being used to train the ML model. Thus, such univariate filters may miss cooperativity effects among features. In principle, wrapper FS techniques, such as Recursive Feature Elimination (RFE) [75], may improve model accuracy by capturing these effects at the risk of identifying a local optimum instead of the globally best solution. However, at least in the related problem of cancer prognosis prediction [76], univariate filters generally outperform RFE despite the computational cost of RFE being much higher. In this same study, embedded (built-in) FS techniques, such as LASSO, did not generally outperform univariate filters either. Instead of using a fixed, predetermined cut-off, e.g., the 100 top-ranked genes as in that study [76], a novel aspect of OMC is that the complexity of the ML model is optimised for the drug, cancer type, molecular profile and available data. As the dimensionality of the employed data is optimally reduced for the considered case, thousands of less informative gene alterations are not included in model building. This is often an advantage, as the least informative of these features are probably irrelevant and hence harm classifier performance.

In practice, we have found that OMC complements the standard version of RF on this type of problem (16 of the 26 cases were better predicted by RF-OMC). More importantly, RF-OMC predicts 11 cases with an MCC of at least 0.3, whereas RF-all only predicts 4 cases with at least that accuracy (see Figure S6). Furthermore, while RF-OMC predicts 21 of the 26 cases better than random, this is only the case for 16 of the 26 cases predicted by RF-all. Interestingly, unlike here with multi-omics features of tumours, FS did not generally result in more accurate ML models when using chemical features of drug molecules in the related problem of QSAR [77]. In our study, RF-all only outperforms RF-OMC once among the most predictive cases (i.e., those with an MCC of at least 0.3) when predicting BRCA PDX response to HDM201 based on SNV profiles. This is probably due to the limitations of this initial version of RF-OMC, where features are pre-ranked without taking into account their possible synergies with the rest of the features, and the cut-off might be too tight. These exceptions are hence a reminder of the importance of considering multiple ML algorithms.

The results also show that the accuracy in predicting in vivo tumour response strongly depends on the treatment, regardless of cancer type. The boxplots in Figures S7 and S8 show that this variability is strongly reduced as we consider more types of molecular profiles and classifiers. Still, MCCs across treatments vary from slightly above 0 to about 0.6 (rightmost boxplot), despite practically the same numbers of profiled PDXs being available for each treatment. These MCCs also mean that the ML approach, even with OMC, fails in some cases. A possible reason for failure is that the optimal profile and/or classifier could not have been found yet for the treatments with worse predictions. For example, profiles probing non-coding genes, miRNAs, DNA methylation or proteomics, not among the available NIBR-PDXE profiles, might provide the basis to predict the response to some treatments better. We, therefore, recommend considering all available profiles for ML modelling. Another reason is that responsive and non-responsive PDXs may form two well-separated clusters in feature space in some treatments but not in others (the former case being easier for classification than the latter). This topology is controlled by multiple convoluted factors, such as data biases (e.g., inter-tumour molecular and response heterogeneity), the choice of representation (e.g., GEX) or how complex is the relationship between response and profile (e.g., drug polypharmacology). Lastly, experimental errors might be larger in some cases. For instance, a different response would be measured if two mice were engrafted with the same tumour, but they metabolised the drug differently.

As anticipating which classifiers will work best on a case is currently not possible, these are benchmarked on a similar case to select those most suitable [78]. Here we benchmarked three algorithms to build models of varying complexity. In this way, their relative performance can shed some light on the intrinsic non-observable complexity of each case. For instance, only a given list of genes could be controlling drug response for tumours of that type, and that control could be preferentially exerted at a given omics level. In the few cases where the single-gene marker worked best, it is likely that such a subset is actually reduced down to that single gene, and the omics level is SNV (e.g., the mutational status of ACR as a predictor of CRC PDX response to cetuximab in Figure 2B). However, it could also be that mutations in other genes influence drug response as well, but we do not have sufficient data to detect more complex cooperativity patterns. In cases where RF-OMC works best, a few genes are likely to be controlling drug response via the most predictive molecular profile (e.g., the mutational status of NR1H2, TLK2 and CTSA to predict BRCA PDX response to LKA136 in Figure 2A), although again more data could result in more complex gene interactions being exploited and thus models with higher predictive accuracy. In cases where RF-all obtains the best performance (e.g., an RF model trained on all SNV features to predict BRCA PDX response to HDM201 in Figure 2A), this suggests that more features than those considered by the OMC strategy must be positively contributing to the prediction of drug response.

### 4.3. Future Application to Clinical Data

While the generalisation of these methodology research outcomes to patient data is still to be confirmed, the fact that they have been observed in both in vitro and in vivo preclinical models is promising. If such generalisation was observed, without generating any additional data, many more patients would benefit from precision oncology by applying multiple ML algorithms to existing clinical pharmacogenomics data (our study has also made a set of data modelling recommendations that can be applied to the analysis of any similar data sets). Moreover, as PDXs capture the diversity and complexity of their originating tumours, the translational potential of the generated predictors in a clinical setting is likely. Improved predictors for paclitaxel and cetuximab, both of which are standards of care for breast and colon cancers, respectively, could hence have an impact on cancer treatment effectiveness in the clinic. Our approach could also be applied to generating improved predictors of response to drugs in development (e.g., binimetinib for BRCA), supporting the use of ML for patient selection in clinical trials. Beyond these potential clinical applications, our study shows that ML can be employed to improve our understanding of cancer biology. On the one hand, ML could anticipate which PDXs not harbouring the actionable mutation are responsive (these would have therefore been missed by existing single-gene markers). On the other hand, the OMC strategy provides a concise list of gene alterations that control treatment response in the considered cancer type (these alterations have been individually linked to cancer hallmarks, therapeutic response and prognosis as explained on pages 7–10 in the Supplementary Information file). Thus, an alternative polygenic hypothesis explaining treatment response can be generated by combining both streams of information.

## 5. Conclusions

This study has proposed a ML approach to combine multiple gene alterations in cancer cells and predict its response to cancer drugs. The results have shown that the ML model better predicts results than the corresponding single-gene markers in 19 of 26 cases. We have also introduced a strategy termed OMC to effectively reduced the dimensionality of pharmacogenomics data by selecting only the features that are important to drug response prediction. Importantly, this ML approach (both standard RF and RF-OMC) can determine the most predictive classifier type and molecular profile for each treatment- cancer type pair. Thus, by evaluating several models (SG, standard RF, RF-OMC) and molecular profile types (CN, CNA, SNV, GEX), the prediction of a given case can be effectively increased. Moreover, the ML classifiers also obtain higher recall than the corresponding SG markers; therefore, many more patients could benefit from precision oncology by applying complementarily multiple ML algorithms than from using only the current SG markers.

Another advantage of RF-OMC over the standard RF model is that this OMC version requires only a concise list of genes to be profiled to predict the sensitivity of a PDX to a cancer drug. This gene list permits cheaper and faster clinical implementation. Furthermore, it can be considered as a promising starting point for further studies in the underlying mechanisms of drug sensitivity in cancer cells. Last but not least, the most predictive RF-OMC models revealed the genes that are not just data-driven but biologically relevant to cancer, as found by other independent experimental studies.

## Figures and Tables

**Table 1:** Random Forest model with Optimal Model Complexity (OMC) and the corresponding single-gene marker in one replicate for the three best cases, including BRCA—binimetinib, BRCA—paclitaxel and CRC—cetuximab. MCC: Matthews Correlation Coefficient; PR: Precision; RC: Recall; F1: F1 score.

Cancer Type	Treatment	RF-OMC					Single-Gene Marker			
		Profile	MCC	PR	RC	F1	MCC	PR	RC	F1
BRCA	binimetinib	GEX	0.57	0.77	0.91	0.83	0.24	0.68	0.68	0.68
BRCA	paclitaxel	SNV	0.49	0.82	0.88	0.85	−0.82	0	0	NA
CRC	cetuximab	SNV	0.47	0.62	0.81	0.70	0.59	0.9	0.56	0.69

**Table 2:** RF model using the randomly selected subset of features for the 26 treatment-cancer type pairs.

Cancer Type	Treatment_Name	Profile	nPDXs	MCC	Precision	Recall	Specificity	F1
BRCA	BGJ398	SNV	38	NA	0.37	1.00	0.00	0.54
BRCA	BKM120	SNV	38	NA	NA	0.03	1.00	NA
BRCA	BYL719	GEX	38	0.02	0.64	0.77	0.29	0.69
BRCA	BYL719 + LJM716	SNV	38	NA	NA	0.02	1.00	NA
BRCA	CGM097	CN	38	−0.17	0.07	0.04	0.88	NA
BRCA	CLR457	CNA	38	NA	NA	0.04	0.95	NA
BRCA	HDM201	SNV	38	NA	0.37	1.00	0.00	0.54
BRCA	INC424	SNV	37	0.03	0.38	0.93	0.11	0.54
BRCA	LEE011	SNV	38	NA	NA	0.07	0.93	NA
BRCA	LKA136	SNV	38	NA	0.32	1.00	0.04	0.48
BRCA	LLM871	CN	38	NA	NA	NA	NA	NA
BRCA	binimetinib	GEX	38	0.08	0.60	0.75	0.31	0.67
BRCA	paclitaxel	SNV	38	NA	NA	0.00	1.00	NA
CRC	BKM120	GEX	39	0.00	0.64	0.82	0.21	0.71
CRC	BYL719	CN	41	0.21	0.70	0.79	0.40	0.73
CRC	BYL719 + LJM716	CNA	39	NA	NA	0.02	0.92	NA
CRC	CGM097	GEX	36	−0.04	0.27	0.18	0.78	NA
CRC	CLR457	CN	40	0.02	0.70	0.89	0.13	0.78
CRC	HDM201	SNV	39	NA	0.32	1.00	0.06	0.48
CRC	LEE011	SNV	41	0.13	0.88	0.08	0.97	NA
CRC	LKA136	CN	39	−0.13	0.25	0.18	0.68	NA
CRC	binimetinib	CN	41	0.02	0.67	0.67	0.32	0.67
CRC	cetuximab	SNV	40	0.19	0.42	1.00	0.15	0.59
CRC	CKX620	CN	38	−0.09	0.76	0.97	0.00	0.85
CRC	encorafenib	CN	40	−0.12	0.11	0.08	0.88	NA
CRC	LFW527 + binimetinib	SNV	31	0.36	0.70	0.63	0.67	0.69

## Data Availability

This study did not generate any data.

## Supplementary Materials

The following are available online at https://www.mdpi.com/article/10.3390/biomedicines9101319/s1. Supplementary Tables: Description of processed NIBR-PDXE data and the discovered multi-omics predictors of in vivo treatment response along with the performance in all 104 analysed data sets (26 cases × 4 profiles). Supplementary Figures: Figure S1: Visualising criteria employed by the NIBR-PDXE to categorise PDX responses to a treatment from the temporal evolution of tumour volume growth, Figure S2: Comparison of RF-OMC versus random predictions for each of the 13 treatments administered to Breast Cancer (BRCA) PDX models, Figure S3: Comparison of RF-OMC versus random predictions for each of the 13 treatments administered to Colorectal Cancer (CRC) PDX models, Figure S4: Comparison of RF-all versus random predictions for each of the 13 treatments administered to BRCA PDX models, Figure S5: Comparison of RF-all versus random predictions for each of the 13 treatments administered to CRC PDX models, Figure S6: Performance comparison of RF-OMC and RF-all models in predicting tumour response, Figure S7: Prediction performance improves as more classifier and profile types are considered for predicting the response of BRCA PDXs to 13 treatments, Figure S8: Prediction performance improves as more classifier and profile types are considered for predicting the response of CRC PDXs to 13 treatments, Figure S9: A version of Figure 2 showing MCC variability across 10 LOOCV repetitions with error bar (each with a different random seed), Figure S10: BRCA- binimetinib whose most predictive model has the expressions of 14 genes as features. Supplementary information: A literature review of the cancer relevance of the selected features from the three most predictive RF-OMC models.
