# Supplementary material for: Predicting Cancer Drug Response In Vivo by Learning an Optimal Feature Selection of Tumour Molecular Profiles
Source: Biomedicines. 2021 Sep 26;9(10):1319. doi: 10.3390/biomedicines9101319 (PMC8533095; doi:10.3390/biomedicines9101319)
Supplement: Supplementary file 1 [file biomedicines-09-01319-s001.zip › biomedicines-supplementary.pdf]

biomedicines

MDPI

Supplementary Information

Predicting Cancer Drug Response In Vivo by Learning an Optimal Feature Selection of Tumour Molecular Profiles

Linh C. Nguyen [1,2], Stefan Naulaerts [3,4], Alejandra Bruna [5], Ghita Ghislat [6] and Pedro J. Ballester [1,*]

1 Cancer Research Center of Marseille, INSERM U1068, F-13009 Marseille, France; nguyencamlinh@hus.edu.vn
2 Department of Life Sciences, University of Science and Technology of Hanoi, Vietnam Academy of Science and Technology, Hanoi 100803, Vietnam
3 Ludwig Institute for Cancer Research, 1200 Brussels, Belgium; stefan.naulaerts@bru.licr.org
4 Duve Institute, UCLouvain, 1200 Brussels, Belgium
5 The Institute of Cancer Research, London SM2 5NG, UK; Alejandra.bruna@icr.ac.uk
6 Centre d'Immunologie de Marseille-Luminy, Inserm, U1104, CNRS UMR7280, F-13009 Marseille, France; gghislat1@gmail.com
* Correspondence: pedro.ballester@inserm.fr; Tel.: + 33-(0)-4-86-97-72-01

Supplementary Figures

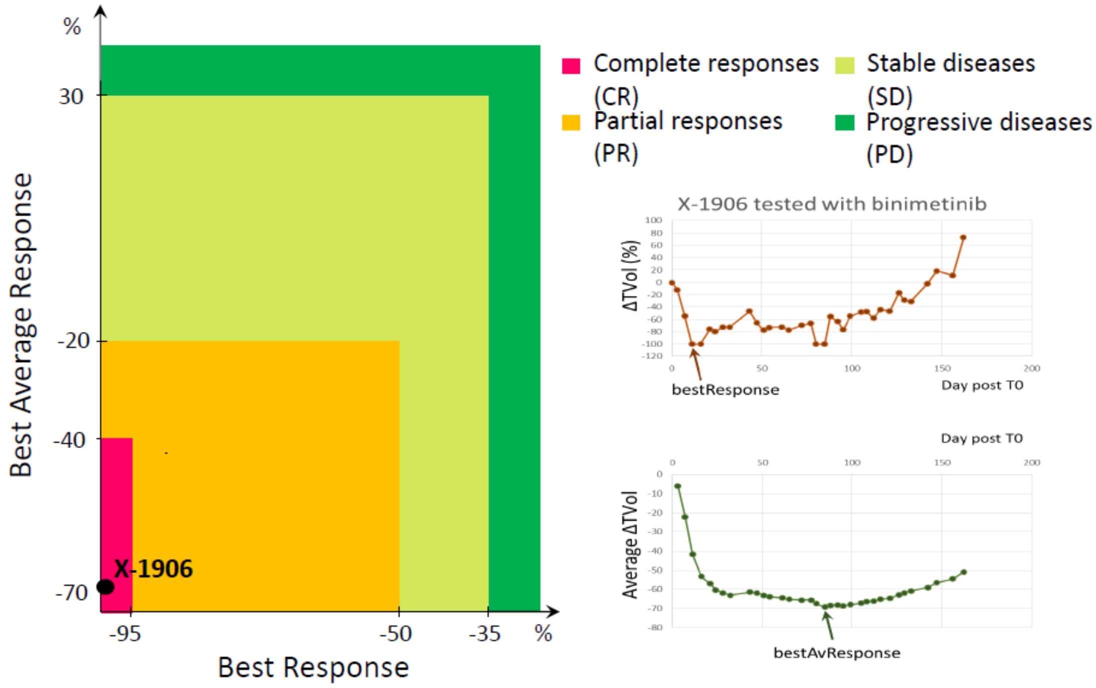

Figure S1. Visualising criteria employed by the NIBR-PDXE to categorise PDX responses to a treatment from the temporal evolution of tumour volume grow. NIBR-PDXE employed a set rules, which and rules are visualised here for clarification purposes, including an example of a PDX with complete remission (X-1906). Both rules and nomenclature above are defined in the Methods section of the paper (e.g. ΔTVol corresponds to the % of variation in tumour volume with respect to initial tumour volume).

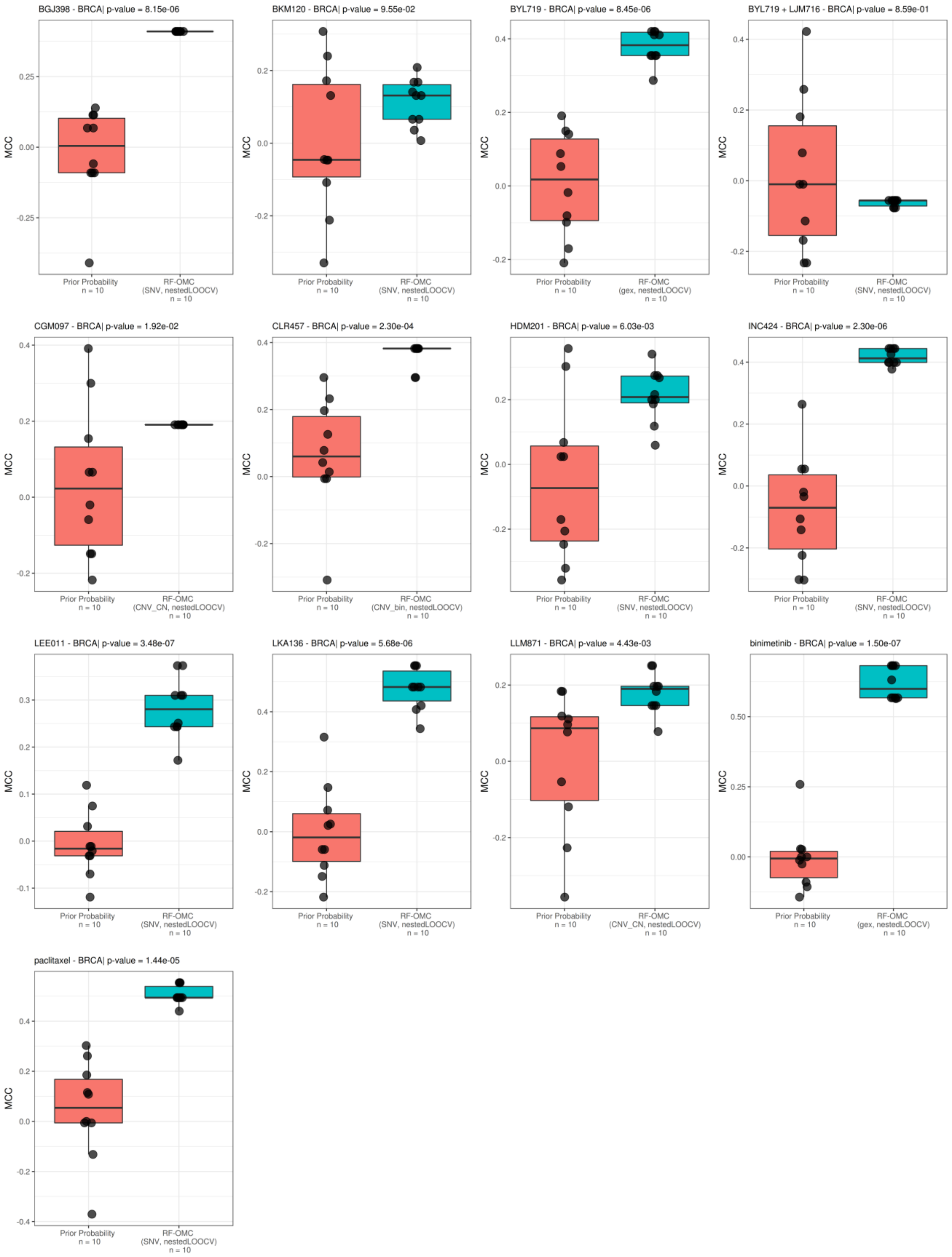

Figure S2. RF-OMC versus random predictions for each of the 13 treatments administered to Breast Cancer (BRCA) PDX models. The random model is based on the prior probabilities for the case (see Methods section). For each of the 13 treatments, a turquoise boxplot summarises the MCCs of the 10 runs of RF-OMC. The magenta boxplot contains the MCCs of the 10 runs of the random model using prior probabilities. Each calculated MCC is presented as black point. From the p-values reported on the title of each plot, we can see that RF-OMC predicts 11 of the 13 treatments significantly better than random (p-value < 0.05; one-sided paired t-test).

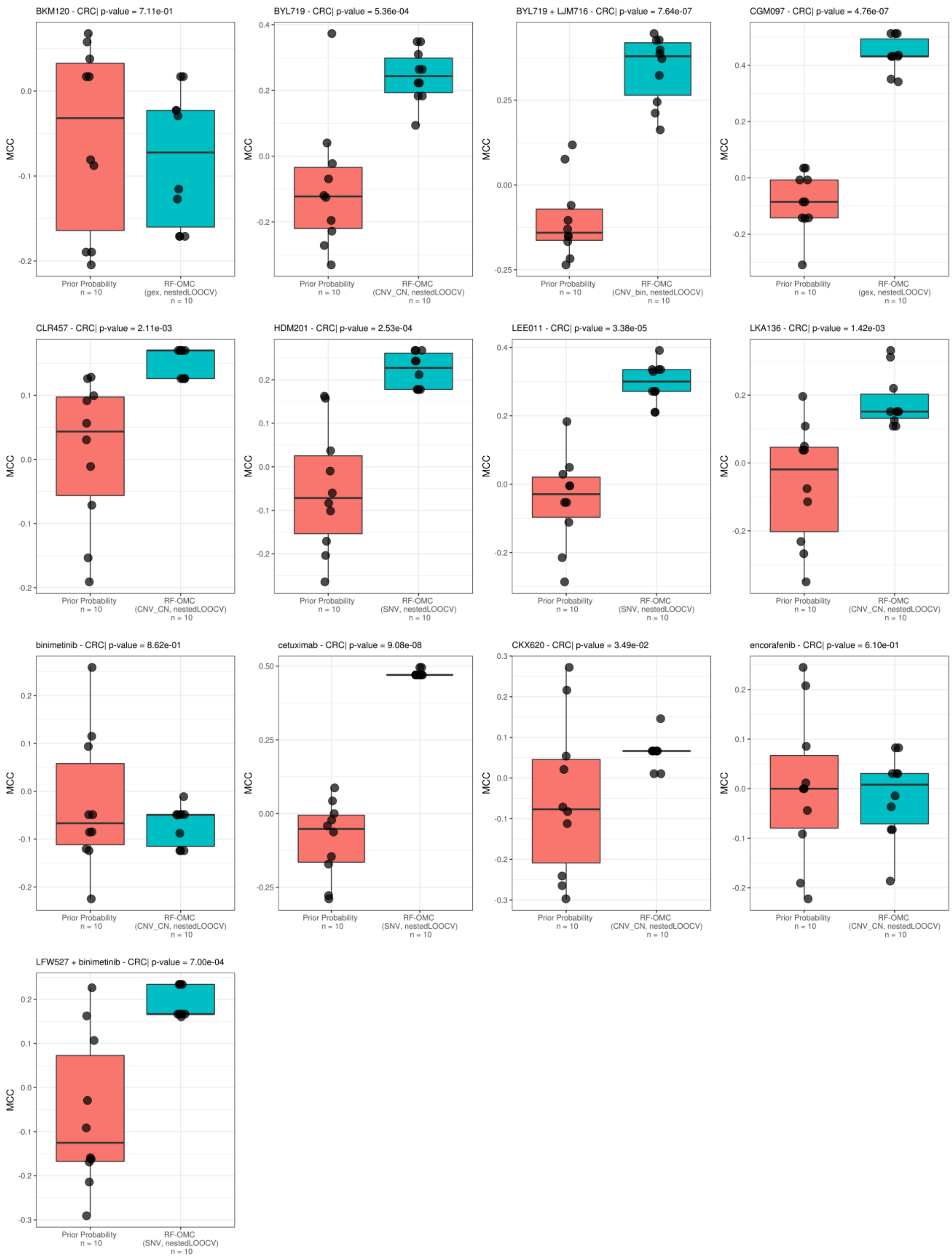

Figure S3. RF-OMC versus random predictions for each of the 13 treatments administered to Colorectal Cancer (CRC) PDX models. The random model is based on the prior probabilities for the case (see Methods section). For each of the 13 treatments, a turquoise boxplot summarises the MCCs of the 10 runs of RF-OMC. The magenta boxplot contains the MCCs of the 10 runs of the random model using prior probabilities. Each calculated MCC is presented as black point. From the p-values reported on the title of each plot, we can see that RF-OMC predicts 10 of the 13 treatments significantly better than random (p-value < 0.05; one-sided paired t-test).

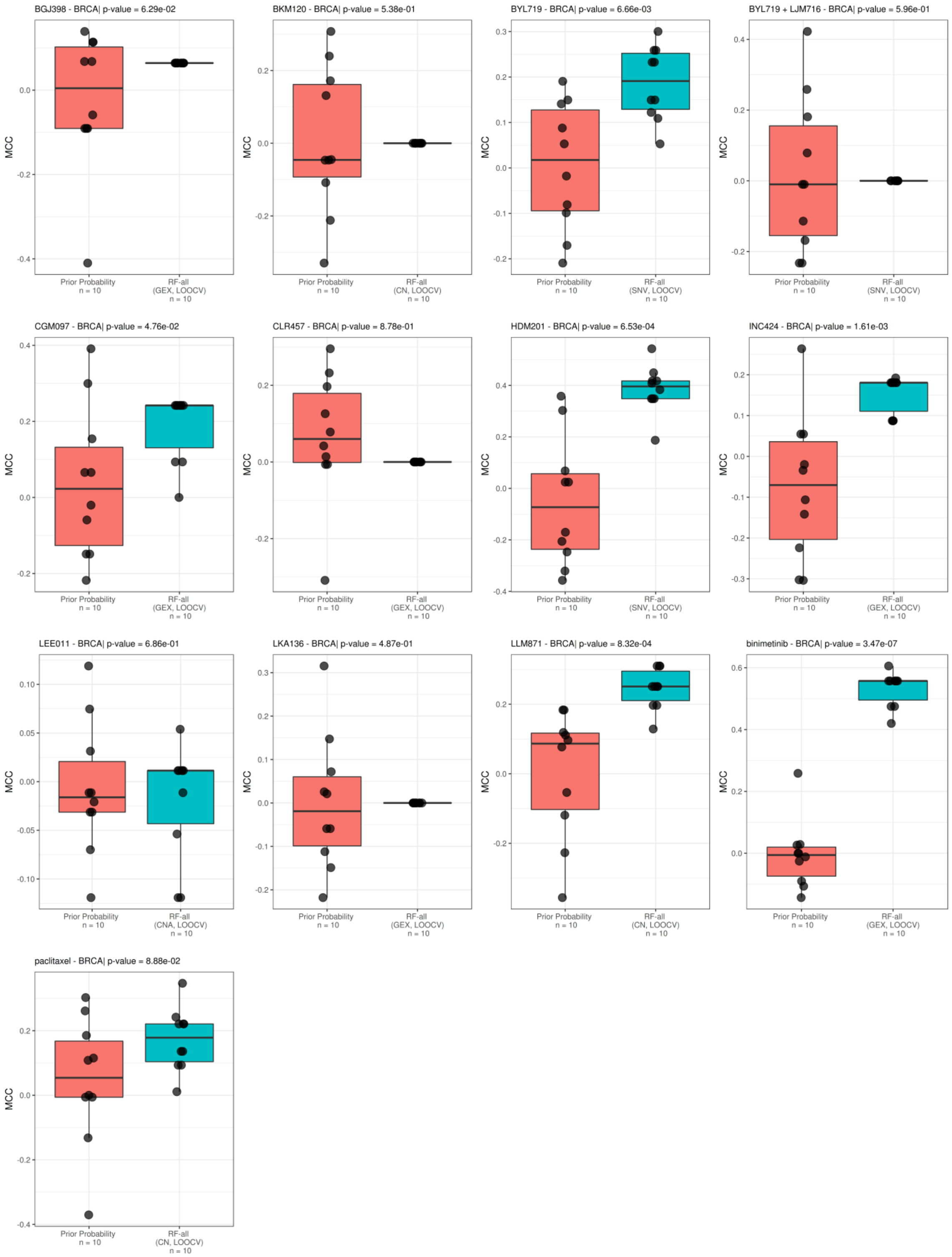

Figure S4. RF-all versus random predictions for each of the 13 treatments administered to BRCA PDX models. The random model is based on the prior probabilities for the case (see Methods section). For each of the 13 treatments, a turquoise boxplot summarises the MCCs of the 10 runs of RF-all. The magenta boxplot contains the MCCs of the 10 runs of the random model using prior probabilities. Each calculated MCC is presented as black point. From the p-values reported on the title of each plot, we can see that RF-all predicts 6 of the 13 treatments significantly better than random (p-value < 0.05; one-sided paired t-test).

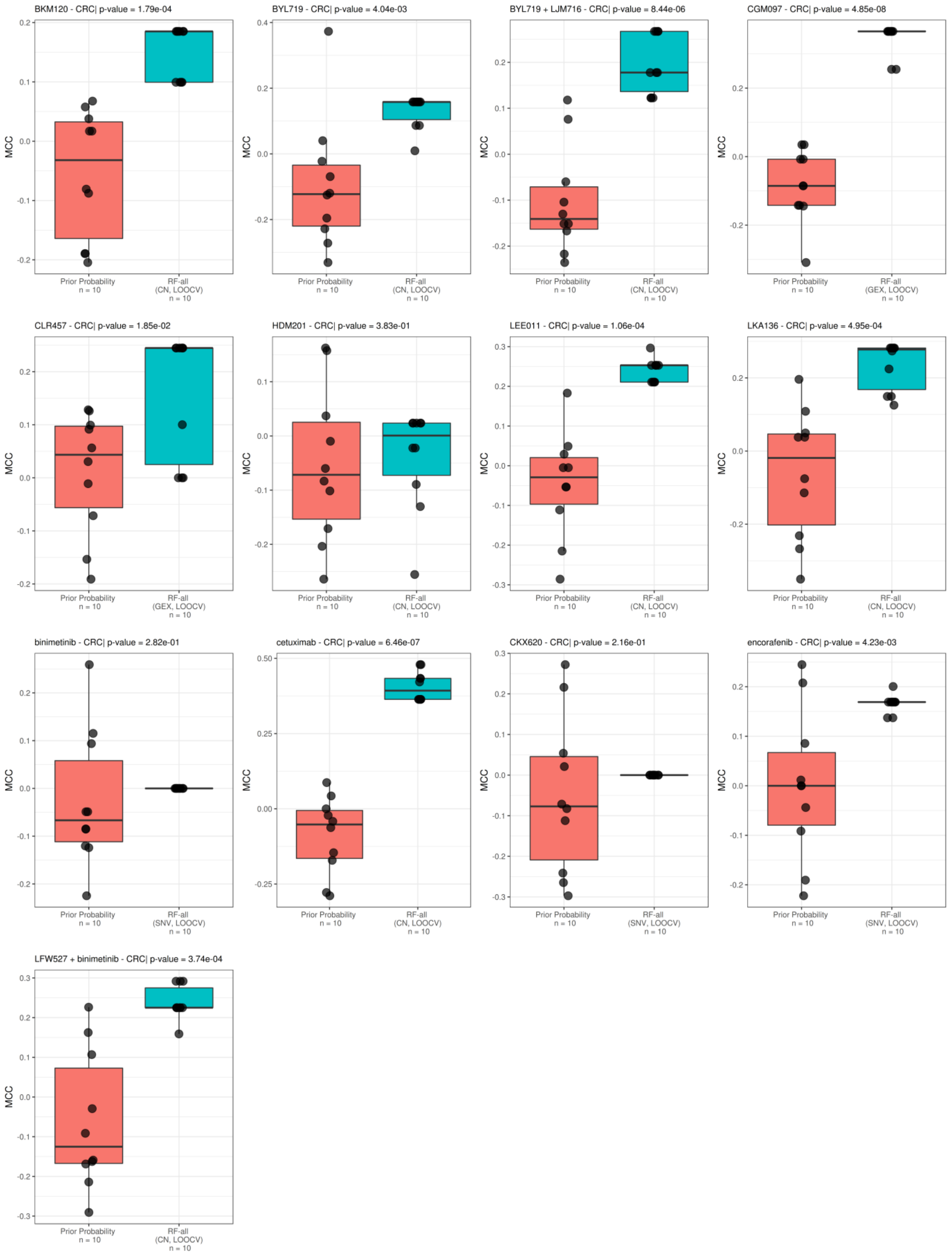

Figure S5. RF-all versus random predictions for each of the 13 treatments administered to CRC PDX models. The random model is based on the prior probabilities for the case (see Methods section). For each of the 13 treatments, a turquoise boxplot summarises the MCCs of the 10 runs of RF-all. The magenta boxplot contains the MCCs of the 10 runs of the random model using prior probabilities. Each calculated MCC is presented as black point. From the p-values reported on the title of each plot, we can see that RF-all predicts 10 of the 13 treatments significantly better than random (p-value < 0.05; one-sided paired t-test).

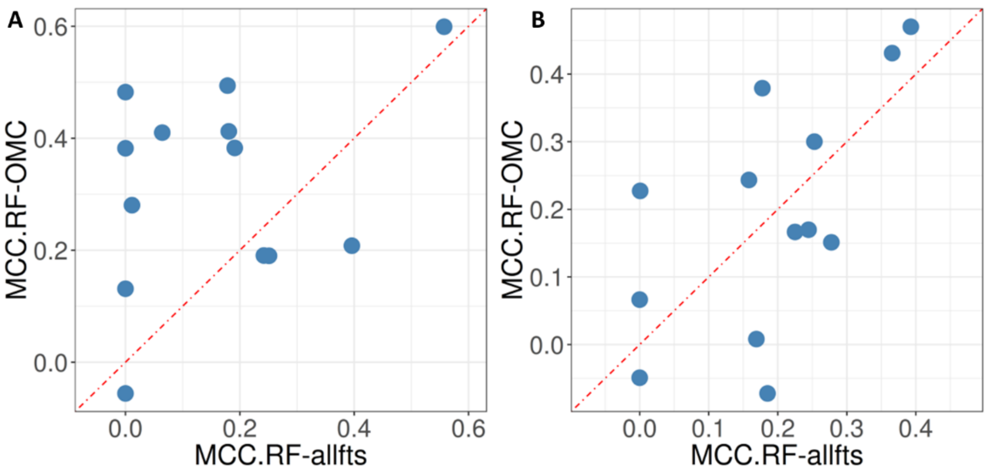

Figure S6. Performance comparison of RF-OMC and RF-all models in predicting tumour response. **(A)** BRCA RF-OMC models achieved better MCC than RF-all models in 9 of 13 treatments tested on BRCA PDXs (7 RF-OMC models with MCC of at least 0.3, for only 2 RF-all models). **(B)** RF-OMC models achieved better MCC than RF-all models in only 7 of 13 treatments tested on CRC PDXs (4 RF-OMC models with MCC of at least 0.3, for only 2 RF-all models).

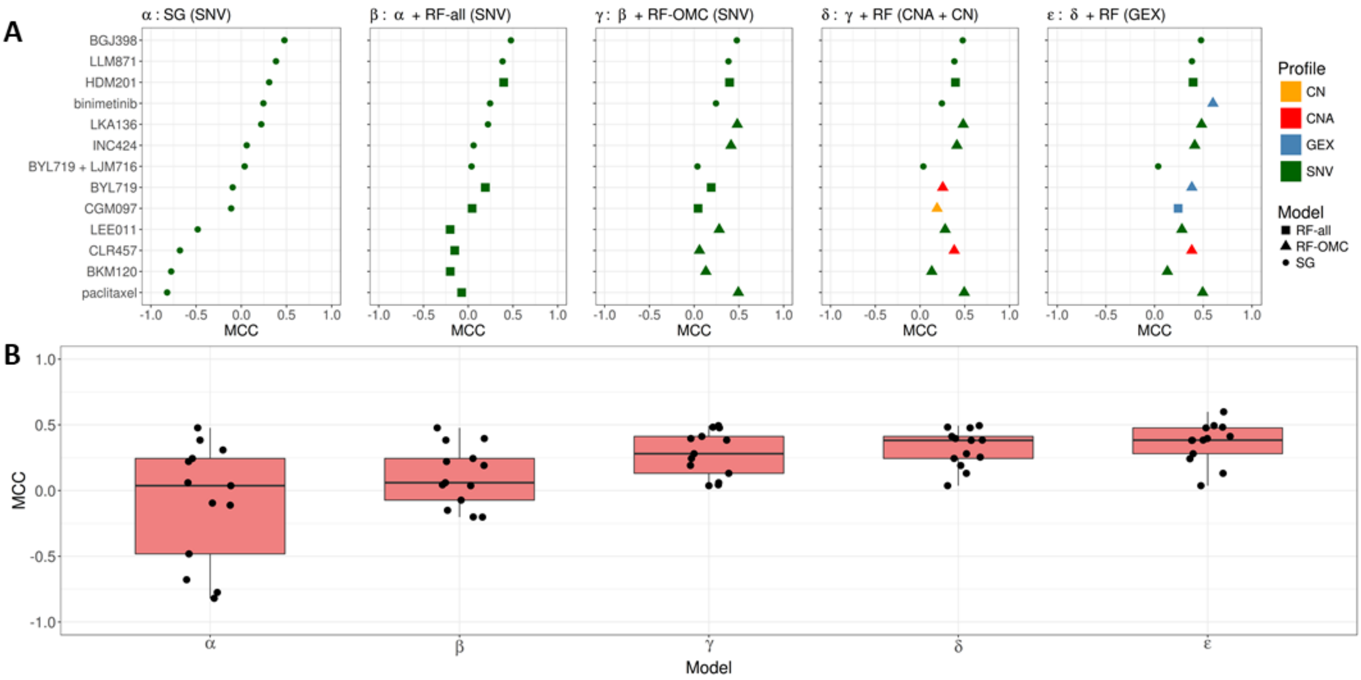

Figure S7. Prediction performance improves as more classifier and profile types are considered for predicting response of BRCA PDXs to 13 treatments. **(A)** In each of these five plots (α to ε), each row shows the cross-validated MCC of the best predictor for that treatment as we consider more classifiers and tumour profiles. **α** – Single-gene (SG) markers using Single-Nucleotide Variant (SNV) data. **β** – Also considering Random Forest model with all features (RF-all) leads to an improvement in prediction performance in 7 out of 13 treatments. Both models (SG and RF-all) considered SNV data. γ – also considering RF-OMC helped increase the accuracy in predicting 6 out of 13 treatments in comparison to plot β. All three models (SG, RF-all and RF-OMC) were trained using SNV data. **δ** – Models considering both SNV and copy-number data, including real-valued (CN) and binary copy-number alterations (CNA). The addition of these profiles results in an increase in performance in 3 out of 13 treatments in comparison to plot γ. **ε** – Models also considering the gene expression (GEX) profiles, the performance is improved in 3 out of 13 treatments compared to plot δ. With all classifiers and available profiles considered, 10 of the 13 treatments can now be predicted with MCC>0.25. By contrast, in the common case of using a single classifier and profile represented by α and using the same PDXs, only 3 treatments

can be predicted with this level of accuracy. **(B)** Each boxplot summarises the MCC values of the employed classifiers across the 13 treatments for each case mentioned above.

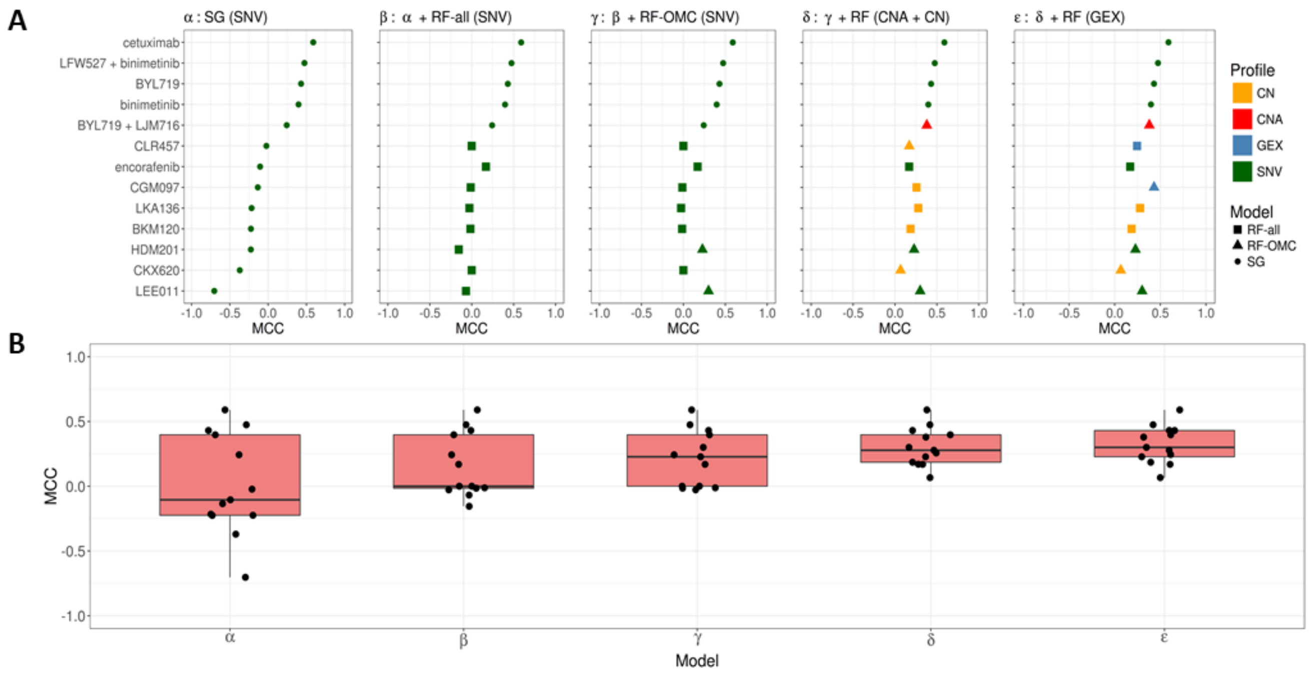

Figure S8. Prediction performance improves as more classifier and profile types are considered for predicting response of CRC PDXs to 13 treatments. **(A)** In each of these five plots (α to ε), each row shows the cross-validated MCC of the best predictor for that treatment as we consider more classifiers and tumour profiles. **α** – Single-gene (SG) markers using Single-Nucleotide Variant (SNV) data. **β** – Also considering Random Forest model with all features (RF-all) leads to an improvement in prediction performance in 8 out of 13 treatments. Both models (SG and RF-all) considered SNV data. **γ** – also considering RF-OMC helped increase the accuracy in predicting 2 out of 13 treatments in comparison to plot β. All three models (SG, RF-all and RF-OMC) were trained using SNV data. **δ** – Models considering both SNV and copy-number data, including real-valued (CN) and binary copy-number alterations (CNA). The addition of these profiles results in an increase in performance in 6 out of 13 treatments in comparison to plot γ. **ε** – Models also considering the gene expression (GEX) profiles, the performance is improved in 2 out of 13 treatments compared to plot δ. With all classifiers and available profiles considered, 8 of the 13 treatments can now be predicted with MCC>0.25. By contrast, in the common case of using a single classifier and profile represented by α and using the same PDXs, only 4 treatments can be predicted with this level of accuracy. **(B)** Each boxplot summarises the MCC values of the employed classifiers across the 13 treatments for each case mentioned above.

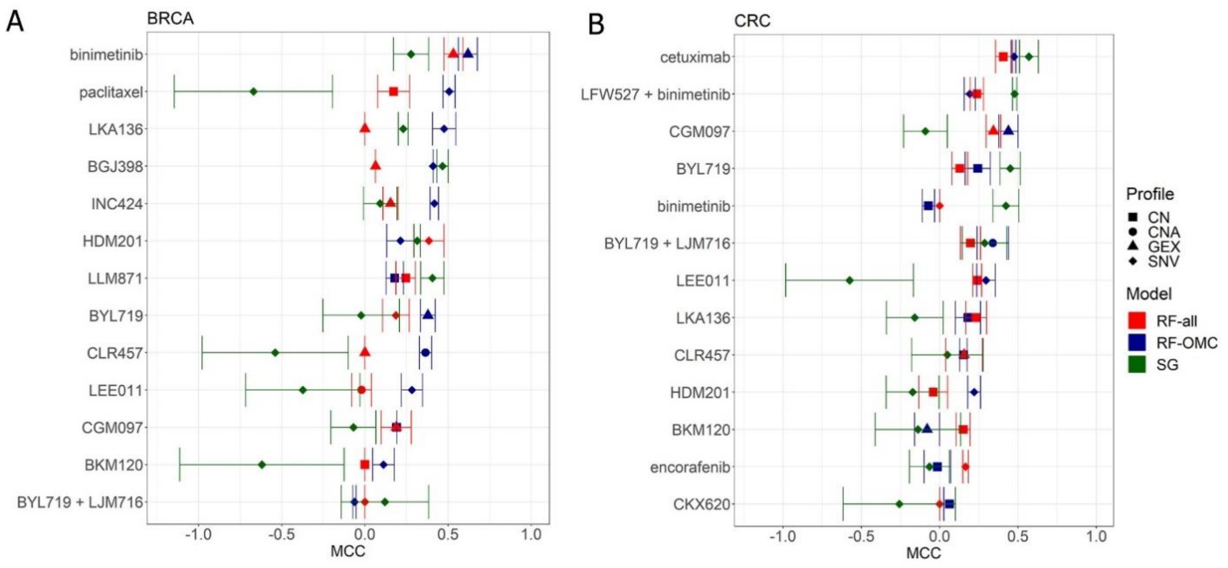

Figure S9: A version of Figure 2 showing MCC variability across 10 LOOCV repetitions (each with a different random seed). Predictive performance of the best single-gene (SG) marker, Random Forest (RF) with Optimal Model Complexity (RF-OMC) and RF using all features (RF-all) per treatment-cancer type pair. For each of these three models, the mean MCC +/- standard deviation of MCC across repetitions is shown, with the colour and shape of each classifier indicating the model type and employed molecular profile, respectively. For instance, binimetinib appears as a blue triangle, which means that binimetinib had RF-OMC-SNV as the classifier with the best mean MCC on LOOCV held-out PDXs. **(A)** BRCA PDXs. **(B)** CRC PDXs.

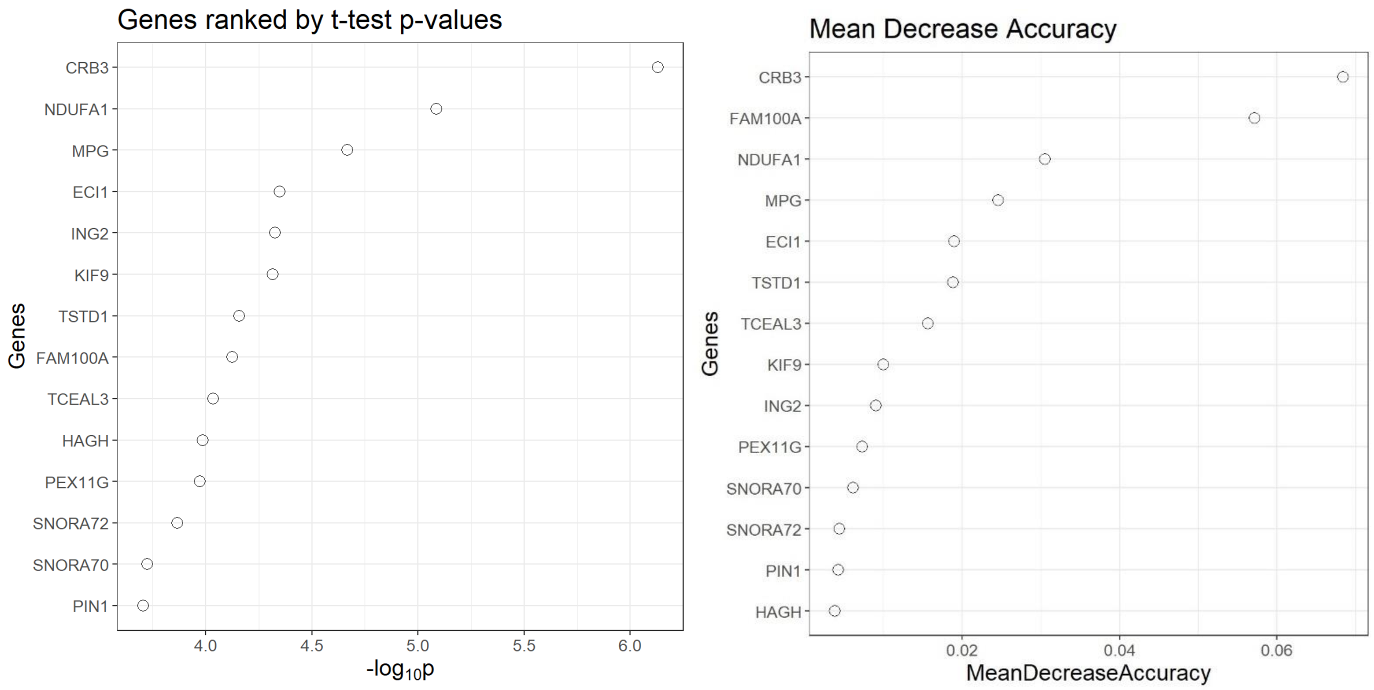

Figure S10: BRCA- binimetinib whose most predictive model has the expressions of 14 genes as features. The ranking of these features by p-value (as in OMC) is similar to that by RF's feature importance (Mean Decrease Accuracy). Note that the most notable exception is FAM100A, which is much highly ranked when taking into account other features.

Supplementary Information

RF-OMC classifier to predict the response of BRCA PDXs to paclitaxel

This model exclusively employs the mutational status of two genes MUC20 and UPK3BL. The MUC20 gene encodes for a member of the mucin protein family. Mucins are high molecular-weight extracellular glycoproteins which are secreted by epithelial cells to form an insoluble mucous barrier. Many mucins have been found to be abnormally expressed or glycosylated in adenocarcinomas as well as associated with carcinogenesis, tumour invasion and poor patient outcome [1]. In particular, the MUC20 gene has also been found to promote aggressive phenotypes in ovarian cancer [2), and to be the predictor of recurrence and poor outcome in CRC [3]. High MUC20 expression is associated with response to chemotherapy in esophageal squamous cell carcinoma [4]. The UPK3BL gene encodes for a protein in the uroplakins protein family. It has been found among the upregulated genes in rhabdoid glioblastoma tumour, suggesting that this gene may be functionally involved in this type of cancer [5].

RF-OMC classifier to predict the response of BRCA PDXs to binimetinib

This model uses the expression levels of just 14 genes: CRB3, NDUFA1, MPG, ECI1, ING2, KIF9, TSTD1, FAM100A, TCEAL3, HAGH, PEX11G, SNORA72, SNORA70 and PIN1. The CRB3 gene encodes for a protein in the CRB protein family, which plays various roles in the control of cytokinesis, ciliogenesis and the formation of tight junctions between cells, as well as involves in the establishment of cell polarity in epithelial cells [6]. Dysregulation of cell polarity proteins could play an important role in cancer development [6]. The overexpression of CRB3 gene has been show to inhibit breast cancer cell growth and promote apoptosis *in vitro*, as well as to reduce tumour growth *in vivo* [7]. Meanwhile, a reduced expression of CRB3 was proved to induce carcinogenesis in mouse kidney epithelial cells [8]. Another study has shown that CRB3 affects the expression of the epithelial-mesenchymal transition (EMT) transcriptional repressor [9]. The EMT process – the biological process in which the polarized epithelium undergoes changes in the cell cytoskeleton, loses epithelial features and acquires mesenchymal characteristics like migration and invasion – is known to play essential roles in cancer development [10]. The MPG gene encodes for a DNA repair enzyme, *N*-methylpurine-DNA glycosylase. This gene has increased expression in breast cancer cell lines in comparison to normal breast epithelial cells [11]. The ING2 gene is a member of the inhibitor of growth [ING] protein family. These proteins function in DNA repair and apoptosis. The expression of ING2 gene has been found significantly reduced in human melanoma, and the under-expression of ING2 could be an important event in the initiation of melanoma development [12]. Other studies have suggested that ING2 gene could be a tumour suppressor gene in the carcinogenesis of head and neck squamous cell cancer [13] and in lung cancer [14]. Although no evidence has been found for the KIF9 gene relating to carcinogenesis or cancer progression, this gene has been shown to be required for chromosome alignment and mitotic progression [15]. The TCEAL3 gene encodes for a protein in the transcription elongation factor A family, which may function as a nuclear phosphoprotein modulating transcription in a promoter-dependent manner. A study on the association between gene expression and drug resistance in colorectal cancer patients discovered that TCEAL3 is one of the genes that significantly associate with patient survival [16]. The HAGH gene encode for the enzyme glycoxylase II in charge of catalyzing the glutathione-dependent metabolism of cytotoxic methylglyoxal, which protects cells against cellular damage and apoptosis [17]. An increasing body of evidence has suggested that glycoxylase II participates in the initiation and progression of urological maglinancies, including prostate cancer [18,19], renal cancer [20,21] and bladder cancer [22]. PEX11G is the gene encoding for the Peroxisomal Biogenesis Factor 11 Gamma – a protein that joins in the regulation of number and sizes of the peroxisomes in the cells. Although no direct association has been found between the PEX11G gene and carcinogenesis, a study pointed out that this gene is one of the direct target of TP53 [23]. SNORA70 and SNORA72 are two members of the

small nucleolar RNA genes. While the roles of these two genes have not been comprehensively studied, certain other members of this gene family were found to act as oncogenes or tumour suppressors genes *in vitro* [24,25]. Last but not least, the gene PIN1 encodes for the Peptidyl-prolyl cis/trans isomerases, which catalyzes the cis/trans isomerization of peptidyl-prolyl peptide bonds. PIN1 plays an important role in breast development, as well as it is a target of several oncogenic pathways and was found overexpressed in breast cancer [26,27]. Besides, the overexpression of PIN1 also promotes *in vitro* cell growth of osteosarcoma cell lines [28].

RF-OMC classifier to predict the response of CRC PDXs to cetuximab

This model considered the gene expression levels of only four genes: ACR, DENND4B, NOTCH1 and RPL22. Interestingly, this model considers the gene NOTCH1, whose association with cancer is well known. This gene encodes a transmembrane receptor participating in the Notch signalling pathway. Aberrations in the genes of this pathway could have a large impact on cellular division leading to cancer [29]. This pathway has been found dysregulated in CRC, where the upregulation of NOTCH1 is associated with poor survival outcome [30]. Although no mutation of this gene has been reported in CRC, a study found that NOTCH1 amplification in metastatic CRC patients could lead to worse survival in this subgroup of patients [31]. The gene RPL22 encodes for a cytoplasmic ribosomal protein that is a component of the 60S subunit. Mutations and abnormal expression of RPL22 gene has been reported in various types of cancers, including T-cell acute lymphoblastic leukemia [32], endometrial cancer [33], colorectal cancer [34], non-small cell lung cancer [35] and gastric cancer[36]. We could not find any study linking ACR or DENND4B to CRC, but the high predictive accuracy of this classifier demonstrates the important role of these genes. Experimental studies intended to unravel the role of these genes is therefore promising.

Among the genes resulted from our multigene RF-OMC classifiers, a high number of them have reported to be associated with cancer in the literature. It is noteworthy that these genes were selected via a data-driven approach. Therefore, these results suggested that the models found by RF-OMC are biologically relevant and can be further investigated for the application in practice.

References

1. Yonezawa S, Goto M, Yamada N, Higashi M, Nomoto M. Expression profiles of MUC1, MUC2, and MUC4 mucins in human neoplasms and their relationship with biological behavior. Proteomics [Internet]. 2008 Aug [cited 2018 Jun 27];8(16):3329–41. Available from: http://www.ncbi.nlm.nih.gov/pubmed/18651706
2. Chen C-H, Shyu M-K, Wang S-W, Chou C-H, Huang M-J, Lin T-C, et al. MUC20 promotes aggressive phenotypes of epithelial ovarian cancer cells via activation of the integrin β1 pathway. Gynecol Oncol [Internet]. 2016 Jan [cited 2018 Jun 27];140(1):131–7. Available from: http://www.ncbi.nlm.nih.gov/pubmed/26616226
3. Xiao X, Wang L, Wei P, Chi Y, Li D, Wang Q, et al. Role of MUC20 overexpression as a predictor of recurrence and poor outcome in colorectal cancer. J Transl Med [Internet]. BioMed Central; 2013 Jun 20 [cited 2018 Jun 27];11:151. Available from: http://www.ncbi.nlm.nih.gov/pubmed/23787019
4. Shen L-Y, Wang H, Dong B, Yan W-P, Lin Y, Shi Q, et al. Possible prediction of the response of esophageal squamous cell carcinoma to neoadjuvant chemotherapy based on gene expression profiling. Oncotarget [Internet]. 2016 Jan 26 [cited 2018 Jun 27];7(4):4531–41. Available from: http://www.ncbi.nlm.nih.gov/pubmed/26673820
5. Koh Y, Park I, Sun C-H, Lee S, Yun H, Park C-K, et al. Detection of a Distinctive Genomic Signature in Rhabdoid Glioblastoma, A Rare Disease Entity Identified by Whole Exome Sequencing and Whole Transcriptome Sequencing. Transl Oncol [Internet]. Neoplasia Press; 2015 Aug [cited 2018 Oct 26];8(4):279–87. Available from: http://www.ncbi.nlm.nih.gov/pubmed/26310374
6. Li P, Mao X, Ren Y, Liu P. Epithelial cell polarity determinant CRB3 in cancer development. Int J Biol Sci [Internet]. Ivyspring International Publisher; 2015 [cited 2018 Oct 30];11(1):31–7. Available from: http://www.ncbi.nlm.nih.gov/pubmed/25552927
7. Mao X, Li P, Wang Y, Liang Z, Liu J, Li J, et al. CRB3 regulates contact inhibition by activating the Hippo pathway in mammary epithelial cells. Cell Death Dis [Internet]. 2017 Jan 12 [cited 2018 Oct 31];8(1):e2546–e2546. Available from: http://www.ncbi.nlm.nih.gov/pubmed/28079891
8. Karp CM, Tan TT, Mathew R, Nelson D, Mukherjee C, Degenhardt K, et al. Role of the Polarity Determinant Crumbs in Suppressing Mammalian Epithelial Tumor Progression. Cancer Res [Internet]. 2008 Jun 1 [cited 2018 Oct 31];68(11):4105–15. Available from: http://www.ncbi.nlm.nih.gov/pubmed/18519669

9. Spaderna S, Schmalhofer O, Wahlbuhl M, Dimmler A, Bauer K, Sultan A, et al. The Transcriptional Repressor ZEB1 Promotes Metastasis and Loss of Cell Polarity in Cancer. Cancer Res [Internet]. 2008 Jan 15 [cited 2018 Oct 31];68(2):537–44. Available from: http://www.ncbi.nlm.nih.gov/pubmed/18199550
10. Yao D, Dai C, Peng S. Mechanism of the Mesenchymal-Epithelial Transition and Its Relationship with Metastatic Tumor Formation. Mol Cancer Res [Internet]. 2011 Dec 1 [cited 2018 Nov 2];9(12):1608–20. Available from: http://www.ncbi.nlm.nih.gov/pubmed/21840933
11. Cerda SR, Turk PW, Thor AD, Weitzman SA. Altered expression of the DNA repair protein, N-methylpurine-DNA glycosylase (MPG), in breast cancer. FEBS Lett [Internet]. No longer published by Elsevier; 1998 [cited 2018 Oct 31];431(1):12–8. Available from: https://www.sciencedirect.com/science/article/pii/S0014579398006978
12. Lu F, Dai DL, Martinka M, Ho V, Li G. Nuclear ING2 expression is reduced in human cutaneous melanomas. Br J Cancer [Internet]. Nature Publishing Group; 2006 Jul 3 [cited 2018 Oct 31];95(1):80–6. Available from: http://www.ncbi.nlm.nih.gov/pubmed/16755297
13. Li X, Kikuchi K, Takano Y. ING Genes Work as Tumor Suppressor Genes in the Carcinogenesis of Head and Neck Squamous Cell Carcinoma. J Oncol [Internet]. Hindawi Limited; 2011 [cited 2018 Oct 31];2011:963614. Available from: http://www.ncbi.nlm.nih.gov/pubmed/21052543
14. Okano T, Gemma A, Hosoya Y, Hosomi Y, Nara M, Kokubo Y, et al. Alterations in novel candidate tumor suppressor genes, ING1 and ING2 in human lung cancer. Oncol Rep [Internet]. 2006 Mar [cited 2018 Oct 31];15(3):545–9. Available from: http://www.ncbi.nlm.nih.gov/pubmed/16465410
15. Andrieu G, Quaranta M, Leprince C, Hatzoglou A. The GTPase Gem and its partner Kif9 are required for chromosome alignment, spindle length control, and mitotic progression. FASEB J [Internet]. 2012 Dec [cited 2018 Nov 2];26(12):5025–34. Available from: http://www.ncbi.nlm.nih.gov/pubmed/22964304
16. Zheng Y, Zhou J, Tong Y. Gene signatures of drug resistance predict patient survival in colorectal cancer. Pharmacogenomics J [Internet]. Nature Publishing Group; 2015 Apr [cited 2018 Nov 2];15(2):135–43. Available from: http://www.ncbi.nlm.nih.gov/pubmed/25179828
17. Antognelli C, Talesa VN. Glyoxalases in Urological Malignancies. Int J Mol Sci [Internet]. Multidisciplinary Digital Publishing Institute (MDPI); 2018 Jan 31 [cited 2018 Nov 2];19(2). Available from: http://www.ncbi.nlm.nih.gov/pubmed/29385039
18. Antognelli C, Del Buono C, Baldracchini F, Talesa V, Cottini E, Brancadoro C, et al. Alteration of glyoxalase genes expression in response to testosterone in LNCaP and PC3 human prostate cancer cells. Cancer Biol Ther [Internet]. 2007 Dec [cited 2018 Nov 2];6(12):1880–8. Available from: http://www.ncbi.nlm.nih.gov/pubmed/18344682
19. Talesa VN, Ferri I, Bellezza G, Love HD, Sidoni A, Antognelli C. Glyoxalase 2 Is Involved in Human Prostate Cancer Progression as Part of a Mechanism Driven By PTEN/PI3K/AKT/mTOR Signaling With Involvement of PKM2 and ERα. Prostate [Internet]. Wiley-Blackwell; 2017 Feb [cited 2018 Nov 2];77(2):196–210. Available from: http://doi.wiley.com/10.1002/pros.23261
20. Antognelli C, Baldracchini F, Talesa VN, Costantini E, Zucchi A, Mearini E. Overexpression of glyoxalase system enzymes in human kidney tumor. Cancer J [Internet]. [cited 2018 Nov 2];12(3):222–8. Available from: http://www.ncbi.nlm.nih.gov/pubmed/16803681
21. Di Ilio C, Angelucci S, Pennelli A, Zezza A, Tenaglia R, Sacchetta P. Glyoxalase activities in tumor and non-tumor human urogenital tissues. Cancer Lett [Internet]. 1995 Sep 25 [cited 2018 Nov 2];96(2):189–93. Available from: http://www.ncbi.nlm.nih.gov/pubmed/7585456
22. Mearini E, Romani R, Mearini L, Antognelli C, Zucchi A, Baroni T, et al. Differing expression of enzymes of the glyoxalase system in superficial and invasive bladder carcinomas. Eur J Cancer [Internet]. 2002 Sep [cited 2018 Nov 2];38(14):1946–50. Available from: http://www.ncbi.nlm.nih.gov/pubmed/12204678
23. Parikh N, Hilsenbeck S, Creighton CJ, Dayaram T, Shuck R, Shinbrot E, et al. Effects of TP53 mutational status on gene expression patterns across 10 human cancer types. J Pathol [Internet]. NIH Public Access; 2014 Apr [cited 2018 Nov 2];232(5):522–33. Available from: http://www.ncbi.nlm.nih.gov/pubmed/24374933
24. Nallar SC, Kalvakolanu D V. Regulation of snoRNAs in cancer: close encounters with interferon. J Interferon Cytokine Res [Internet]. Mary Ann Liebert, Inc.; 2013 Apr [cited 2018 Nov 2];33(4):189–98. Available from: http://www.ncbi.nlm.nih.gov/pubmed/23570385
25. Gong J, Li Y, Liu C, Xiang Y, Li C, Ye Y, et al. A Pan-cancer Analysis of the Expression and Clinical Relevance of Small Nucleolar RNAs in Human Cancer. Cell Rep [Internet]. 2017 Nov [cited 2018 Nov 2];21(7):1968–81. Available from: https://linkinghub.elsevier.com/retrieve/pii/S2211124717315334
26. Wulf G, Ryo A, Liou Y-C, Lu KP. The prolyl isomerase Pin1 in breast development and cancer. Breast Cancer Res [Internet]. BioMed Central; 2003 [cited 2018 Nov 2];5(2):76–82. Available from: http://www.ncbi.nlm.nih.gov/pubmed/12631385
27. Rustighi A, Zannini A, Campaner E, Ciani Y, Piazza S, Del Sal G. PIN1 in breast development and cancer: a clinical perspective. Cell Death Differ [Internet]. Nature Publishing Group; 2017 [cited 2018 Nov 2];24(2):200–11. Available from: http://www.ncbi.nlm.nih.gov/pubmed/27834957
28. ZHOU L, PARK B-H, PARK JH, JANG KY, PARK HS, WAGLE S, et al. Overexpression of the prolyl isomerase PIN1 promotes cell growth in osteosarcoma cells. Oncol Rep [Internet]. 2013 Jan 30 [cited 2018 Nov 2];29(1):193–8. Available from: http://www.ncbi.nlm.nih.gov/pubmed/23129219
29. Vinson KE, George DC, Fender AW, Bertrand FE, Sigounas G. The Notch pathway in colorectal cancer. Int J Cancer [Internet]. Wiley-Blackwell; 2016 Apr 15 [cited 2018 Nov 2];138(8):1835–42. Available from: http://doi.wiley.com/10.1002/ijc.29800

30. Chu D, Zhang Z, Zhou Y, Wang W, Li Y, Zhang H, et al. Notch1 and Notch2 have opposite prognostic effects on patients with colorectal cancer. Ann Oncol [Internet]. Oxford University Press; 2011 Nov 1 [cited 2018 Nov 2];22(11):2440–7. Available from: https://academic.oup.com/annonc/article-lookup/doi/10.1093/annonc/mdq776
31. Arcaroli JJ, Tai WM, McWilliams R, Bagby S, Blatchford PJ, Varella-Garcia M, et al. A NOTCH1 gene copy number gain is a prognostic indicator of worse survival and a predictive biomarker to a Notch1 targeting antibody in colorectal cancer. Int J cancer [Internet]. NIH Public Access; 2016 Jan 1 [cited 2018 Nov 2];138(1):195–205. Available from: http://www.ncbi.nlm.nih.gov/pubmed/26152787
32. Rao S, Lee S-Y, Gutierrez A, Perrigoue J, Thapa RJ, Tu Z, et al. Inactivation of ribosomal protein L22 promotes transformation by induction of the stemness factor, Lin28B. Blood [Internet]. 2012 Nov 1 [cited 2018 Nov 2];120(18):3764–73. Available from: http://www.ncbi.nlm.nih.gov/pubmed/22976955
33. Novetsky AP, Zighelboim I, Thompson DM, Powell MA, Mutch DG, Goodfellow PJ. Frequent mutations in the RPL22 gene and its clinical and functional implications. Gynecol Oncol [Internet]. 2013 Mar [cited 2018 Nov 2];128(3):470–4. Available from: http://www.ncbi.nlm.nih.gov/pubmed/23127973
34. Ferreira AM, Tuominen I, van Dijk-Bos K, Sanjabi B, van der Sluis T, van der Zee AG, et al. High Frequency of *RPL22* Mutations in Microsatellite-Unstable Colorectal and Endometrial Tumors. Hum Mutat [Internet]. 2014 Dec [cited 2018 Nov 2];35(12):1442–5. Available from: http://www.ncbi.nlm.nih.gov/pubmed/25196364
35. Yang M, Sun H, Wang H, Zhang S, Yu X, Zhang L. Down-regulation of ribosomal protein L22 in non-small cell lung cancer. Med Oncol [Internet]. 2013 Sep 25 [cited 2018 Nov 2];30(3):646. Available from: http://www.ncbi.nlm.nih.gov/pubmed/23797773
36. Nagarajan N, Bertrand D, Hillmer AM, Zang Z, Yao F, Jacques P-É, et al. Whole-genome reconstruction and mutational signatures in gastric cancer. Genome Biol [Internet]. 2012 Dec 13 [cited 2018 Nov 2];13(12):R115. Available from: http://www.ncbi.nlm.nih.gov/pubmed/2323766
